# The Recent Trends of Systemic Treatments and Locoregional Therapies for Cholangiocarcinoma

**DOI:** 10.3390/ph17070910

**Published:** 2024-07-08

**Authors:** Abdullah Esmail, Mohamed Badheeb, Batool Wael Alnahar, Bushray Almiqlash, Yara Sakr, Ebtesam Al-Najjar, Ali Awas, Mohammad Alsayed, Bayan Khasawneh, Mohammed Alkhulaifawi, Amneh Alsaleh, Ala Abudayyeh, Yaser Rayyan, Maen Abdelrahim

**Affiliations:** 1 Section of GI Oncology, Houston Methodist Neal Cancer Center, Houston Methodist Hospital, Houston, TX 77030, USA; 2 Department of Internal Medicine, Yale New Haven Health, Bridgeport Hospital, Bridgeport, CT 06610, USA; 3 College of Medicine, Almaarefa University, Riyadh 11597, Saudi Arabia; 4 Zuckerman College of Public Health, Arizona State University, Tempe, AZ 85287, USA; bushrayalmiqlash11@gamil.com; 5 Department of GI Medical Oncology, Division of Cancer Medicine, The University of Texas MD Anderson Cancer Center, Houston, TX 77030, USA; 6 Faculty of Medicine and Health Sciences, University of Science and Technology, Sanaa P.O. Box 15201-13064, Yemen; 7 Prolato Clinical Research Center, Houston, TX 77054, USA; 8 Department of Medicine, Desert Regional Medical Center, Palm Springs, CA 92262, USA; 9 Division of Internal Medicine, The University of Texas MD Anderson Cancer Center, Houston, TX 77030, USA; 10 Department of Gastroenterology & Hepatology, Faculty of Medicine, The University of Jordan, Amman 11942, Jordan

**Keywords:** cholangiocarcinoma, hepatic malignancy, intrahepatic (iCCA), perihilar (pCCA), distal (dCCA)

## Abstract

Cholangiocarcinoma (CCA) is a hepatic malignancy that has a rapidly increasing incidence. CCA is anatomically classified into intrahepatic (iCCA) and extrahepatic (eCCA), which is further divided into perihilar (pCCA) and distal (dCCA) subtypes, with higher incidence rates in Asia. Despite its rarity, CCA has a low 5-year survival rate and remains the leading cause of primary liver tumor-related death over the past 10–20 years. The systemic therapy section discusses gemcitabine-based regimens as primary treatments, along with oxaliplatin-based options. Second-line therapy is limited but may include short-term infusional fluorouracil (FU) plus leucovorin (LV) and oxaliplatin. The adjuvant therapy section discusses approaches to improve overall survival (OS) post-surgery. However, only a minority of CCA patients qualify for surgical resection. In comparison to adjuvant therapies, neoadjuvant therapy for unresectable cases shows promise. Gemcitabine and cisplatin indicate potential benefits for patients awaiting liver transplantation. The addition of immunotherapies to chemotherapy in combination is discussed. Nivolumab and innovative approaches like CAR-T cells, TRBAs, and oncolytic viruses are explored. We aim in this review to provide a comprehensive report on the systemic and locoregional therapies for CCA.

## 1. Introduction

**Figure 1:**
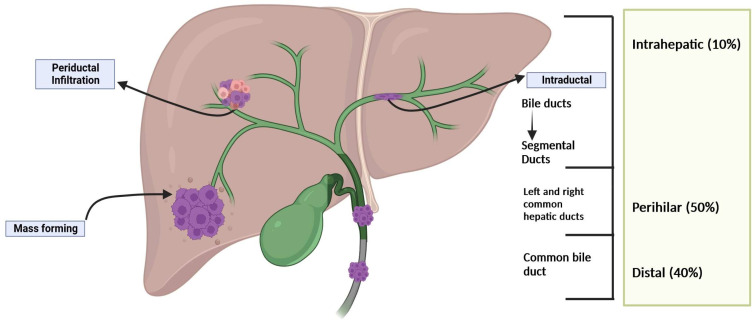
Anatomical classification of cholangiocarcinoma (CCA) based on its origin.

**Figure 2:**
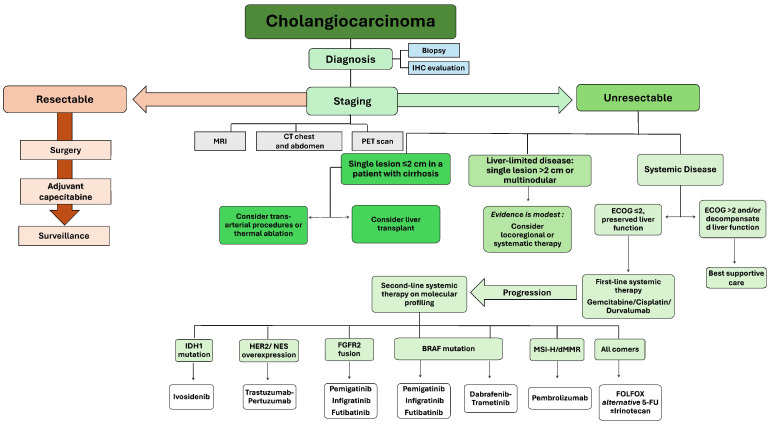
A comprehensive diagram of cholangiocarcinoma treatment options. IHC—immunohistochemistry, MRI—magnetic resonance imaging, CT—computed tomography, PET—positron emission tomography, ECOG—Eastern Cooperative Oncology Group, 5-FU—fluorouracil, dMMR-deficient DNA mismatch repair, MSI-H—microsatellite instability high.

Cholangiocarcinoma (CCA), a rare hepatic malignancy with a rapidly increasing incidence, accounts for less than 5% of total gastrointestinal malignancies. CCA can be divided, based on its origin, into intrahepatic (iCCA), accounting for (10%), and extrahepatic (eCCA), which is further subdivided into perihilar (pCCA), interchangeably known as hilar or Klatskin’s tumor, representing 50%, and distal (dCCA) representing 40% of all cases (Figure 1) [1,2]. In addition to the anatomical classification, pCCA can be grouped into five subtypes based on the involvement of hepatic ducts (Figure 2) [3].

CCA incidence shows a considerable geographic variation; for instance, the reported incidence is 0.3–2/100,000 cases per annum in rich countries, whereas a higher incidence is reported in Asian countries (e.g., China and Thailand), reaching up to 6–7/100,000 cases annually [4,5,6]. Despite its low incidence, in the last three decades, the CCA incidence has increased six-fold [7,8,9]. A nationwide analysis in the United States reported an overall incidence of 1.26/100,000 annually [10]. The worldwide mortality rates of iCCA and eCCA manifest diverse epidemiological trends. Comprehensive global mortality data for iCCA and eCCA are presented in Table 1.

CCA is more frequently observed in patients with conditions such as liver cirrhosis and alcoholic liver disease in Western countries [11]. Additionally, patients with primary sclerosing cholangitis (PSC), especially in the context of ulcerative colitis, tend to have an earlier onset of CCA [12]. Other risk factors include parasitic infection with *Opisthorcis viverini* and *Chlonorchis sinensis*, exposure to nitrosamines from salted fish and fermented fish sauce, and hepatolethiasis especially in Eastern Asian countries [13]. Additional risk factors include viral Hepatitis B and C, HIV, and EBV [14], as well as exposure to carcinogenic agents like the currently band carcinogenic agent Thorotrast and vinyl chloride. Conditions such as choledochal cysts and Caroli disease are also risk factors. Furthermore, nonalcoholic steatohepatitis (NASH) has been reported as a risk factor and a prognostic factor for iCCA; recently, De Lorenzo et al. reported that NASH was found in more than 20% of patients who would have been classified as having no classical risk factors [15]. Finally, nonalcoholic fatty liver disease (NAFLD) and NAFLD-related metabolic conditions can also be a risk factor for the development of primary liver cancer including CCA in a limited proportion of individuals [16]

Despite medical advancement, CCA remains a fatal disease, and the leading cause of primary liver tumor-related death in the past 10–20 years. Surveillance, epidemiology, and end results have reported an overall 5-year survival of 24% and 2% in localized and distant metastasis cases, respectively [17]. A Romanian study reported an overall survival (OS) of 5.5% in 133 patients with CCA, showing a marginal improvement in survival with therapy [18]. In this article, we aim to review the available medical treatments of CCA and assess their effectiveness in extending patient survival.

## 2. Systemic Therapy for CCA

Recently, chemotherapy has been used more extensively for advanced CCA cases. The selection of agents is primarily based on patients’ overall status, tolerance, and agents’ availability (Figure 2) [19,20,21]. The Eastern Cooperative Oncology Group Performance Status (ECOG PS) is classically utilized to assess performance status, which has proven to have both prognostic and predictive utility [22,23]. Nonetheless, gemcitabine-based regimens remain the standard of care for CCA; classically, gemcitabine with cisplatin (GC) has shown superiority over gemcitabine alone with no significant increase in toxicity [24]. Furthermore, S-1, an oral fluoropyrimidine combination of tegafur, gimeracil, and oteracil, has shown favorable efficacy in several clinical studies [25,26,27]. Even in a more recent randomized controlled trial (RCT), S-1 in combination with gemcitabine (GS) appeared to be non-inferior, well-tolerated, and associated with fewer adverse events profiles compared to GC [28]. Furthermore, adding S-1 to GC significantly improved the OS compared to GC in a randomized phase III trial of (KHBO1401-MITSUBA) [29]. Additionally, the ABC-06 trial, which involved 162 cases with advanced biliary tract malignancies, indicated a substantial, although moderate, survival advantage for oxaliplatin-based combination treatment compared to active symptom management alone following the first GC regimen failure (median 6.2 versus 5.3 months) [30]. Moreover, Glimelius et al. observed survival benefits and quality of life improvement with fluorouracil (FU)-based systemic chemotherapy usage in 37 patients with CCA [31].

Recently, based on the results of the TOPAZ-1 trial, both the Food and Drug Administration (FDA) and European Medicines Agency (EMA) have given their approval for gemcitabine with cisplatin plus durvalumab as the new first-line standard treatment for patients with previously untreated advanced biliary tract cancer. The trial involved 685 patients who were randomly assigned to receive either durvalumab (*n* = 341) or a placebo (*n* = 344) alongside chemotherapy, and the study showed significant improvements in overall survival, progression-free survival, and the objective response rate with the combination of durvalumab, gemcitabine, and cisplatin compared to chemotherapy alone. The 24-month overall survival rate of 24.9% with durvalumab plus chemotherapy, compared to 10.4% with chemotherapy alone, suggests a notable long-term survival benefit [32]. Considering these findings, durvalumab in combination with gemcitabine and cisplatin could be seen as the preferred first-line treatment option for patients with advanced biliary tract cancer (BTC) [32]. Different first-line regimens are summarized in Table 2.

### 2.1. Targeted Therapy

As cancer treatment evolves, molecular profiling plays an increasingly crucial role in managing patients with advanced cancer. It helps to identify specific genetic abnormalities and prognostic indicators, guiding the use of targeted therapies for a more precise approach to treatment. In the realm of advanced CAA, the emergence of inhibitors targeting fibroblast growth factor receptor (*FGFR*) fusions and isocitrate dehydrogenase (*IDH*) mutations is reshaping patient care. Looking ahead, potential targets for future “Precision Medicine” strategies in BTC may extend to chromatin remodeling genes like *ARID1*, *BAP1*, and *PBRM1*, as well as other anomalies such as *BRAF* and *RNF43* mutations, amplifications in HER2 and HER3, or fusion events involving *NTRK* genes [52].

Recent advances in gene sequencing and molecular profiling have significantly improved our understanding of mutations associated with CCA. Gene mutations linked to CCA have seen substantial updates since 2020. In 2022, collaborative efforts led to the identification of new genes associated with CCA, as presented in Table 3, including their occurrence rates and associations [53,54,55,56,57]. Our understanding in 2020 was limited to a set of gene mutations, with *FGFR2* and *IDH1* mutations being the most prevalent. However, by 2022, we observed not only a shift in the prevalence of previously identified mutations but also the emergence of new players, such as *IDH2*, TMB high, and MDM, in the context of CCA. Alongside well-known mutations like *KRAS*, *TP53*, and *CDKN2A*, other genetic alterations are recognized as having significant roles in CCA tumorigenesis. Regarding the pathogenesis of CCA, *FGFR2* abnormalities have been identified in up to 16% of CCA patients, and up to 20% of iCCA cases harbor *FGFR2* alterations [58,59,60,61]. Mutations in the *IDH* genes, particularly *IDH1* and *IDH2*, are found in up to 25% and approximately 3% of CCA cases, with a higher incidence in iCCA cases. Specific inhibitors like ivosidenib and enasidenib are available as a targeted therapy for these mutations [62]. Patients with neurotrophic tyrosine receptor kinase (*NTRK*) gene rearrangements may benefit from *TRK* inhibitors, though these mutations are relatively rare in iCCA [59,63]. *BRAF* gene mutations, particularly *BRAF V600E*, are reported in about 3% of biliary tract cancers, especially iCCA.

This transformation in our understanding of CCA genetics suggests a more nuanced and complex genetic basis for the disease. With this evolving knowledge, targeted genetic testing for advanced CCA cases can provide the foundation for individualized and precise therapeutic approaches.

Notably, patients with *FGFR2* fusion transcript cases and those with an *FGFR2* mutation showed dramatic responses to therapeutic *FGFR* inhibition, consistent with other reported favorable results targeting *FGFR* mutations/fusions in advanced CCA [58,64,65]. Other promising targets for biliary tract cancers include mutations in *IDH1* and *IDH2*, for which specific inhibitors like ivosidenib and enasidenib are available, as well as the BRAF V600E mutation [62,66]. Importantly, the mere presence of a potentially targetable mutation does not guarantee a favorable treatment response. In cases where applicable, consideration should be given to enrolling in a clinical trial for molecularly targeted but unapproved treatments.

- ***IDH1* mutations** are present in around 10–20% of CCA cases and are responsible for the accumulation of oncometabolites, which leads to epigenetic changes affecting various signaling pathways. The FDA has approved ivosidenib for treating adult patients with unresectable or metastatic *IDH1*-mutated CCA as detected by an FDA-approved test, specifically in cases where the disease has progressed after one to two prior lines of systemic therapy for advanced disease. This approval was based on data from the phase III ClarIDHy study, a double-blind placebo-controlled trial. Ivosidenib, the first *IDH1* inhibitor, has shown significant improvements in both PFS (2.7 vs. 1.4 months) and OS (10.3 vs. 5.1 months [adjusted median OS]) compared to placebo in patients with chemorefractory IDH1-mutated CCA [67].
- ***HER2* overexpression:** In 15–20% of extrahepatic CCA and gallbladder carcinomas, there is observed overexpression or amplification of the *EGFR* family receptor tyrosine-protein kinase erbB-2 (*HER2*) [21]. Several investigations into *HER2* alterations’ targeting initially yielded discouraging findings. Between 2021 and 2023, a total of eleven studies examined outcomes from seven distinct *HER2*-targeted medications in BTC. Among these, one of the most recent trials, the phase II trial NCT02091141, explored trastuzumab with pertuzumab. The study enrolled 39 patients, resulting in an ORR of 23% (95% CI 11–329) and a median OS of 10.9 months (95% CI 5.2–15.6) [68]. The phase II open-label ROAR trial demonstrated a 47% objective response rate with the BRAF inhibitor dabrafenib combined with trametinib [69,70]. Up to 20% of CCA, particularly dCCA, have amplifications of *HER2*, and accumulating evidence supports the benefit of *HER2*-targeted therapies, such as trastuzumab plus pertuzumab or lapatinib, or trastuzumab deruxtecan, in such cases [71,72,73].
- ***FGFR2* fusions:** Mutations in *FGFR2* are present in 13–20% of CCAs, often involving activating translocations through fusion or rearrangements. These mutations drive increased cell proliferation, metastasis, and angiogenesis. Pemigatinib received FDA approval for the treatment of adults facing previously treated, unresectable locally advanced or metastatic CCA [74]. This approval targets patients with an *FGFR2* fusion or rearrangement, as identified through an FDA-approved test. The approval was obtained through the FIGHT-202 (NCT02924376) trial, a multicenter open-label single-arm study. Within this trial, 107 patients with locally advanced unresectable or metastatic CCA, whose disease had progressed post at least one prior therapy, were examined. These patients exhibited an ORR of 36% (95% confidence interval: 27–45), with a median DOR of 9.1 months. Moreover, the FDA granted approval to infigratinib, an inhibitor targeting FGFR1–3. This approval was primarily influenced by its favorable outcomes in a phase II trial (NCT02150967). In this trial, patients with previously treated advanced CCA demonstrated an ORR of 23.1%. The trial revealed a median DoR of 5 months and a median PFS of 7.3 months [21].
  On the other hand, futibatinib received accelerated approval from the FDA to address the treatment needs of adult patients confronting previously treated, unresectable, locally advanced, or metastatic iCCA exhibiting *FGFR2* fusions or other rearrangements. This decision stemmed from the study TAS-120–101, a multicenter open-label, single-arm trial. Notably, the median DoR in this trial was recorded at 9.7 months [75].
- ***BRAf* mutation:** In the phase II “VE basket” trial, patients aged 18 years or older with *BRAFV600E*-mutated BTC, whether it was unresectable, metastatic, locally advanced, or recurrent, were included. Over a median follow-up of 10 months, 22 out of 43 patients achieved an investigator-assessed overall response, yielding a response rate of 51% (95% CI 36–67). Additionally, the median overall survival (mOS) was 14 months (95% CI 10–33). These promising outcomes led to the FDA approval of the dabrafenib and trametinib combination for treating advanced *BRAF V600E*-mutated solid malignancies in patients who had undergone prior therapy but experienced progression [76].
- **MSI-H:** Pembrolizumab has received FDA approval as a treatment for patients facing metastatic or inoperable solid tumors characterized by high microsatellite instability (MSI) or mismatch repair (MMR) deficiency. This approval is supported by findings from two pivotal studies: KEYNOTE-158 (NCT02628067) and KEYNOTE-028 (NCT02054806) [77].
- Second-line therapy initiation is contingent upon the tumor’s mutational profile, as delineated in the targeted therapy section. For patients lacking actionable alterations or facing contraindications to targeted treatment, chemotherapy remains the preferred treatment modality. In contrast to first-line chemotherapy, very limited RCTs support an optimal second-line regimen. Moreover, there is no well-established stratification or selection system for second-line therapy candidates. Among some possible regimens, short-term infusional FU plus leucovorin (LV) and oxaliplatin (FOLFOX) is an active regimen for second-line therapy, particularly in patients initially treated with GC [78,79,80]. Danmei Zhang and colleagues conducted a systematic review study, which revealed that while not formally approved, FOLFOX is widely recognized as a standard second-line therapy, supported by findings from the British phase III ABC-06 study [81]. Nevertheless, ongoing debate surrounds whether commencing oxaliplatin-based therapy immediately following cisplatin failure represents the most effective approach, considering both mechanism of action and toxicity. Data from a randomized phase II trial unveiled no discernible difference in OS between FOLFOX and 5-FU, LV, and irinotecan (FOLFIRI). However, the toxicity profiles of these regimens exhibited notable disparities. Neuropathy and thrombocytopenia were more prevalent in the FOLFOX arm, while vomiting and cholangitis were more common in the FOLFIRI arm. Alternatively, the South Korean NIFTY study showcased the superior efficacy of the combination of liposomal irinotecan and 5-FU compared to 5-FU alone [81].

Other second-line therapies include antiangiogenic medications, which act by inhibiting vascular endothelial growth factor (*VEGF*), which is overexpressed in up to half of biliary cancers and has been associated with poorer outcomes [82]. Another second-line option for metastatic iCCA is bevacizumab in combination with FOLFIRI, which has revealed promising efficacy and safety [83]. In another study, bevacizumab combined with erlotinib showed a clinical benefit in advanced biliary malignancies [84]. However, no clinical trials have yet proved bevacizumab’s possible benefits. Regorafenib, on the other hand, showed promising results with improved progression-free survival (PFS) and disease control rate (DCR) in two clinical trials [85,86]. Nevertheless, the data regarding antiangiogenic therapy are sparse, and prospective studies are required to evaluate their efficacy. Table 4 summarizes various targeted therapies for the most prevalent mutations in CCA.

### 2.2. Immunotherapies

Immunotherapy has exhibited promise in several solid malignancies, and in the context of CCA, immune checkpoint inhibitors are particularly valuable for malignancies characterized by DNA with deficient mismatch repair (dMMR)/high microsatellite instability (MSI), programmed cell death 1 ligand 1 (PD-L1) overexpression, or high levels of tumor mutational burden (TMB-high) [107,108].

The introduction of immunotherapies alongside the GC regimen has shown promise in patients with advanced biliary tract cancer. The TOPAZ phase III trial evaluated the combination of GC with or without durvalumab and tremelimumab as a first-line treatment for advanced biliary tract cancer. The results were encouraging, with 66% of patients achieving an objective response with this combination therapy. Grade 3 and 4 adverse events were manageable, and no unexpected safety concerns arose. These findings suggest that adding immunotherapy to the GC regimen could offer a new treatment avenue with promising efficacy and acceptable safety for biliary tract cancer patients [36]. These advancements emphasize the need to explore new treatment strategies, including the addition of targeted therapies and immunotherapy to existing regimens, to improve outcomes for patients with this challenging disease [21].

There is also emerging evidence supporting their use in treatment-naïve iCCA cases with undetermined biomarker status [109]. Notably, the KEYNOTE-158 trial revealed that pembrolizumab, a PD-1 inhibitor, performed poorly with an ORR of 5.8% in advanced biliary tract malignancies [109]. In contrast, nivolumab, another PD-1 inhibitor, demonstrated efficacy in previously treated biliary tract cancer patients, exhibiting an ORR of 22% and a median OS of 14 months in a phase II trial [110]. A separate phase II trial investigated nivolumab in combination with ipilimumab, a cytotoxic T lymphocyte-associated protein 4 (CTLA4) inhibitor, showing OS and PFS rates of 5.7 months and 2.9 months, respectively [59]. Despite concerns about immune-mediated adverse events due to concurrent hepatic impairment and the risk of biliary obstruction in CCA patients, the phase I/II CheckMate 040 trial reported comparable results in terms of transaminases’ elevation for patients with HCC and other solid tumors treated with nivolumab [111].

Chimeric antigen receptor T-cell therapy (CAR-T) has recently gained recognition as an innovative approach for advanced hematological malignancies. While CAR-T therapy has shown efficacy in hematological malignancies, its outcomes in solid tumors, including CCA, may differ due to variations in the tumor microenvironment [112]. For instance, CAR–epidermal growth factor receptor (EGFR) T-cell therapy was explored as a treatment for advanced biliary tract malignancies harboring EGFR mutations, yielding promising results with a CR and SD observed in patients, along with a median PFS of 4 months [113]. However, the application of CAR-T therapy in clinical practice might be restricted due to limited availability, high costs, and insufficient safety data. T-cell-redirecting bispecific antibodies (TRBAs), effective in hematological malignancies and solid tumors, function by directing CD3 T cells to target cancerous cells. These antibodies have shown enhanced T-cell cytotoxicity and prolonged survival, overcoming cellular exhaustion in various studies [114,115]. Although their application in CCA remains unproven, further studies are needed to determine their role. Recent advances in oncolytic virus and vaccine therapies have facilitated the recruitment of T cells to eliminate virus-infected tumor cells. However, only Talimogene laherparepvec has been approved in the US thus far [116]. In CCA, a few preclinical studies involving measles and other survivin-based conditionally replicative adenoviruses vaccines have indicated potential efficacy, although their actual performance remains uncertain [117,118].

## 3. Neoadjuvant Therapy for CCA

Locally advanced, unresectable iCCA cases often receive neoadjuvant therapy in preparation for potential resection (Figure 2). In a study by Nelson et al., 45 patients undergoing concurrent chemoradiotherapy (CRT) for CCA were examined, with 12 of them receiving neoadjuvant therapy. This approach resulted in complete pathologic responses and complete (R0) resections in three and eight patients, respectively [119]. Neoadjuvant therapy is also considered for patients with PSC or small (3 cm) local hilar CCA before undergoing liver transplantation [120,121,122]. An insightful meta-analysis assessed the impact of neoadjuvant therapy on survival post-liver transplantation, revealing 1-year, 3-year, and 5-year survival rates of 71%, 48%, and 32%, respectively. Furthermore, the recurrence rate was notably lower at 24% for those receiving neoadjuvant therapy compared to 57% without it [123]. Yadav et al. conducted a study using the National Cancer Database, demonstrating that neoadjuvant chemotherapy significantly improved the OS of CCA patients compared to adjuvant chemotherapy. The neoadjuvant group exhibited a median OS of 40.3 months, while the adjuvant group showed 32.8 months. Notably, neoadjuvant therapy resulted in 1-year and 5-year survival rates of 85.8% and 42.5%, outperforming the 84.6% and 31.7% rates associated with adjuvant therapy [124].

In a recent prospective case series of 10 patients, GC combination neoadjuvant therapy displayed promising results for unresectable iCCA or pCCA with no extrahepatic involvement. Patients received a median of 181 days of neoadjuvant therapy before liver transplantation, with 90% experiencing no recurrence or metastasis [122,125,126,127,128]. The OS rates at 1 and 2 years were both 100%, with a continued strong performance at 3 to 5 years, maintaining a 75% survival rate [129]. Additionally, another study that compared patients receiving the GC combination to those on alternative regimens highlighted the improved OS of the former group [127]. While these results are promising, the OS for high-risk CCA patients remains challenging. Addressing this challenge, Maithel et al. investigated a neoadjuvant regimen utilizing gemcitabine, cisplatin, and nab-paclitaxel (GAP) in a phase II trial for resectable, high-risk CCA patients. The trial results demonstrated the feasibility and safety of neoadjuvant GAP treatment, achieving a 73% completion rate for both chemotherapy and surgery, with no treatment-related mortality. The median recurrence-free survival reached 7.1 months, and the OS extended to 24 months for the entire cohort, with OS not being reached for patients who underwent surgical resection [130].

## 4. Adjuvant Therapy for CCA

Local relapse is the most typical form of recurrence following total surgical resection in dCCA and pCCA [131,132]. Distant metastases are comparatively less common in hilar CCA than in gallbladder cancer, although they are still observed. Notably, around 40% of hilar CCA patients experienced a distant recurrence, a lower rate compared to the 85% recurrence rate seen in gallbladder cancer patients [132]. Another study reported that 60% of hilar CCA patients who underwent microscopically complete (R0) resection eventually developed distant metastases [131]. Research indicates the potential benefits of adjuvant therapy for these patients, suggesting a positive impact on their overall treatment outcomes. Additionally, the efficacy of adjuvant therapy using S-1 has been verified in a randomized phase III trial with a significant improvement in survival which suggested adjuvant S-1 could be considered a standard of care for resected biliary tract cancer in Asian patients [133,134].

A meta-analysis by Horgan et al. demonstrated a statistically significant survival benefit (OR 0.53, 95% CI 0.39–0.72) associated with adjuvant therapy [135]. Furthermore, the American Society of Clinical Oncology (ASCO) recommends adjuvant therapy for resectable CCA [136]. Various treatment regimens have been explored, and no single regimen has shown clear superiority. Additionally, these regimens can be supplemented with radiotherapy (RT). For example, the Southwest Oncology Group trial endorsed using capecitabine and gemcitabine, followed by RT and concurrent capecitabine, as a well-tolerated and feasible option [137]. Some studies have even investigated gemcitabine, capecitabine, and LV-modulated FU as monotherapy [138,139]. A significant study conducted across specialized hepato-pancreato-biliary centers in the UK assessed the impact of adjuvant capecitabine on OS in patients with resected biliary tract cancer, including CCA. While the intention-to-treat analysis did not meet its primary endpoint of improving OS, the pre-specified sensitivity and per-protocol analyses suggested that adjuvant capecitabine following surgery could enhance OS in these patients [139]. Nevertheless, the choice of adjuvant therapy remains widely empirical, and further research is needed to validate the effectiveness of each regimen.

## 5. Interventional Therapy

### 5.1. Intra-Arterial Therapies

For patients diagnosed with unresectable iCCA featuring either exclusive hepatic involvement or a predominant hepatic disease manifestation, locoregional therapies emerge as a prospective therapeutic modality within the framework of multimodal treatment (Figure 2) [140,141]. Although CCA is considered a less vascular tumor compared to HCC, the findings derived from several studies demonstrate a survival advantage associated with interventional therapies employing a vascular approach in cases of unresectable CCA [142,143,144,145]. Hepatic arterial infusion chemotherapy (HAIC), conventional transarterial chemoembolization (TACE), drug-eluting bead transarterial chemoembolization (dbTACE), and yttrium-90 radioembolization (Y90-RE) are examples of arterially targeted interventions. These interventions are typically employed for palliative purposes in cases of unresectable CCA; however, they also offer the potential for disease control. Methodological challenges in investigating these modalities arise from the inherent scarcity of CCA cases, compounded by the subset of patient’s ineligible for resection, thereby resulting in a relatively limited cohort for applying each non-curative treatment modality [141].

#### 5.1.1. Hepatic Arterial Infusion Chemotherapy (HAIC)

A HAIC pump permits the administration of chemotherapeutics to the liver directly with higher intra-arterial doses while preserving non-neoplastic tissue viability through the portal vein blood supply [146,147,148]. A further advantage of this approach includes limited systemic toxicity of chemotherapy as it is eliminated via hepatic first-pass metabolism [148]. HAIC has been studied extensively for hepatic malignancies as their advancement renders the patient’s illness unresectable. Its use, however, in unresectable CCA has been reviewed in small clinical trials. For example, the Cantore et al. trial showed an OS of 13.2 months and an overall response of 40% in 25 patients with iCCA who were treated with epirubicin, cisplatin, and continuous FU infusion [149]. Another study by Ghiringhelli et al. included 12 unresectable iCCA cases who were treated with HAIC of 1000 mg/m^2^ gemcitabine followed by systemic oxaliplatin and reported 66% and 91% for the overall response and disease control, respectively. The median PFS and OS were 9.2 months (95% CI: 5.1–29.0) and 9.2 months (CI 13.2–49.7), respectively [150]. Floxuridine, a precursor of FU with a higher potency, is used in HAIC and is superior for attaining a higher concentration than systemic delivery [151,152]. S-1 is an oral fluoropyrimidine intended to enhance the therapeutic potential of 5-FU while reducing its toxicity. Twelve patients who received 5-FU with cisplatin were evaluated for efficacy by Higaki et al. showing a median OS of 10.1 months [153]. Currently, prospective trials are ongoing, which will provide more prospective results of different chemotherapeutic regimens for unresectable iCCA, including the GEMOXIA-02 (NCT03364530) and HELIX ICC (NCT04251715) trials.

#### 5.1.2. Conventional Transcatheter Arterial Chemoembolization (TACE)

As a palliative modality, TACE may provide a good result in managing unresectable iCCA [143,154,155]. In contrast to systemic chemotherapeutic infusion, TACE can deliver a higher concentration directly toward the tumor cells, limiting the systemic toxicity [156]. The chemotherapy agents are emulsified with Lipiodol and delivered through the hepatic artery [157]. Park et al. study included 155 patients and showed tumor regression in 23% of the patients in the TACE arm with survival benefits (12.2 months vs. 3.3 months, *p* < 0.001) [142]. Moreover, Kiefer et al. reported a median survival from the time of diagnosis of 20 months, with a 75% survival rate at 1 year, 39% at 2 years, and 17% at 3 years [158]. Li et al. study revealed a median OS of 63 months among TACE-receiving cases following surgical resection versus 18 months in the non-TACE group (*p* = 0.041) [159]. The role of TACE in hilar CCA was recently evaluated by Zheng et al. using gemcitabine and cisplatin-based embolization in addition to RT, which showed a more extended OS period of 20 months versus 10.5 months in the control group (*p* < 0.05) [160].

#### 5.1.3. Drug-Eluting Bead-Transcatheter Arterial Chemoembolization (dbTACE)

As mentioned earlier, chemotherapeutics is emulsified with Lipiodol to be delivered intra-arterially to the hepatic or biliary tumors. This process, however, may decrease the efficacy and/or increase the systemic side effects [161]. The use of microspheres, rather than Lipiodol, has been shown to provide a more tolerated, safer modality with a better delivery of chemotherapy in unresectable HCC [162]. However, dbTACE for CCA was evaluated in merely a few studies. A report by Poggi et al. in patients with iCCA treated with dbTACE containing oxaliplatin and gemcitabine versus chemotherapy revealed a 44% and 56% PR rate and stable disease (SD), respectively. The median OS was 30 months (vs. 12.7 months in the non-dbTACE group, *p* = 0.004) [163]. Luo et al. reported that 8%, 59.5%, and 67.6% of patients with iCCA achieved a complete response (CR), partial response (PR), and overall response rate (ORR), respectively, with the use of dbTACE [164]. Interestingly, Hori et al. reported a case with advanced iCCA who was treated with dbTACE and had excellent local tumor control without further or concurrent therapies; the patient lived for more than four years [165]. Although these findings support dbTACE use in advanced iCCA, little is known about its role in pCCA.

#### 5.1.4. Radioembolization (RE)

Yttrium-90 radioembolization (Y90-RE) provides an alternate therapeutic option for patients with hepatic and biliary tumors [166]. Utilizing microspheres allows for precise tumor targeting and the preferential delivery of radiation to malignancies while minimizing normal tissue involvement. Although the benefits of this approach are well-established for liver tumors, its application in CCA is less documented [167,168,169]. In a study conducted by Ibrahim et al., which included 24 patients with iCCA treated with Y90-RE, encouraging outcomes were observed. Specifically, 27% achieved PRs, 68% maintained SD, and only 5% experienced disease progression. The median OS was 14.9 months, highlighting the therapeutic potential of Y90-RE for unresectable iCCA [170].

A recent investigation by Sarwar et al. focused on the neoadjuvant Y90-RE in iCCA and yielded a DCR of 86% with a median PFS of 5.4 months. Importantly, 52% of iCCA patients treated with a neoadjuvant approach underwent resections, and the majority achieved R0 margins, underscoring the potential of Y90-RE as a neoadjuvant therapy option for unresectable iCCA patients [171]. Furthermore, Chan et al. explored the combination of selective internal radiation therapy using yttrium-90 microspheres, followed by GC chemotherapy for unresectable iCCA. Their findings demonstrated promising outcomes, with a median OS of 13.6 months for the entire cohort and 21.6 months for patients receiving chemotherapy. The response rate reached 25%, and the DCR was 75%, accompanied by manageable adverse events. This approach highlights the feasibility and effectiveness of combining selective internal radiation therapy with standard chemotherapy for unresectable iCCA, offering potential benefits to patients [172]. The existing data support the view that Y90-RE is an efficient, well-tolerated approach for aggressive iCCA cases, with a response rate ranging from 24% to 82.3% [163,164,173,174].

### 5.2. Ablation

Various ablative methods are discussed in the literature, and radiofrequency ablation is one of the most researched ablation methods. It employs rapid vibration-induced frictional heat, resulting in tissue necrosis [175]. Esophageal and primary or malignant hepatic tumors have been managed with radiofrequency ablation (RFA). RFA is often associated with a shorter hospitalization period, reduced treatment costs, and a reduced risk of complications compared to surgery [176,177]. Furthermore, survival benefits were shown in a meta-analysis by Han with RFA use in unresectable iCCA, with a pooled 1-year survival rate of 82% [178]. Despite the promising effects of RFA, the survival benefits are influenced by tumor size in multiple studies [179,180,181]. RFA efficacy was evaluated by Wu et al. for dCCA; compared to controls, the RFA arm had an extended stent patency period, superior functional status, and enhanced quality of life (*p* = 0.001) [182].

The alternative non-thermal option includes irreversible electroporation (IRE), which induces cellular death by disrupting the lipid bilayer [183,184,185]. This technique is relatively recent, with minimal studies. Belfiore et al. evaluated the safety profile, practicality, and effectiveness of IRE in managing CCA, involving a total of 15 cases with unresectable CCA, revealing a reduction in the tumor’s total volume and a subsequent drop in its densitometric values. The median survival time was 18 months [186]. Martin et al. observed an increase in catheter-free days in advanced hilar CCA patients with obstructive jaundice who had undergone IRA [187]. Moreover, Franken et al. reported a 21-month median OS from diagnosis, with a 1 y survival rate of 75% observed following IRE [188]. Another method using microwave ablation was well-tolerated in treating iCCA using a minimally invasive technique. According to Owen, et al. 2023, the median OS for iCCA patients treated with microwave ablation varies from 8.8 to 31.5 months, and the median PFS ranges from 6.2 to 18.43 months [189]. Lastly, cryoablation, which induces cellular death through altering cellular osmotic pressure and dehydration [190], was evaluated by Glazer et al. in a study that included 299 hepatic tumors with an efficacy rate of 89.5%, yet CCA compromised only 6 (2%) of the total cases. Thus, there is insufficient evidence to compel the use of cryoablation in CCA [191]. Currently, a clinical trial is ongoing to evaluate cryoablation in conjunction with anti-PD1 antibodies in patients with advanced iCCA (NCT04299581).

### 5.3. Radiation Therapy (RT)

Radio-chemotherapy serves as an essential adjunctive approach for managing patients with unresectable, locally advanced, or metastasized CCA. In cases of locally advanced CCA, RT can be administered to patients with a favorable performance status. While prospective trials assessing the role of RT in CCA are limited, RT has demonstrated potential benefits, including the alleviation of symptoms such as pain and biliary obstruction [192]. An analysis involving 48 patients with extrahepatic malignancies over a 20-year period (1998–2018) found that a median radiation dose of 50.4 Gy led to a median OS of 12 months. The study revealed an OS of 33% at 2 years, 20% at 3 years, and 7% at 5 years post-therapy [193]. The effectiveness of 125I brachytherapy in patients with unresectable primary and metastatic hepatic malignancies, though primarily focused on HCC, also shows promise for iCCA [194]. Additionally, 125I has demonstrated the potential to prevent stent restenosis, as indicated by a study by Zhu et al., which reported reduced restenosis rates at 3, 6, and 12 months [195]. Furthermore, 125I seed-loaded biliary stents have shown extended patency and OS and provided improved prognoses when compared to conventional separate biliary stents [196,197,198]. Intra-luminal brachytherapy, as briefly reviewed by Khosla et al., represents a tumor-selective alternative that minimizes the impact on normal structures compared to external beam radiation [199]. Moreover, the STRONG study, involving six patients with unresectable pCCA, implemented chemotherapy followed by stereotactic body radiation therapy. The results indicated a median local control rate of 80% with minimal adverse events, suggesting stereotactic body radiation therapy as a safe and viable palliative option [200].

Recent research has delved into the role of RT in addressing advanced liver cancers, particularly challenging cases like unresectable CCA and iCCA. High-dose, hypofractionated proton beam therapy has emerged as a potential solution for these complex-to-treat malignancies. One study involving patients with unresectable HCC and iCCA has demonstrated encouraging outcomes, highlighting the high local control rates achievable with proton therapy and its potential to enhance outcomes in advanced liver cancers [201]. In a separate study, 66 patients with unresectable iCCA received HF-RT using proton or photon RT. The findings revealed a substantial 84% local control rate and a 58% OS at the two-year mark, with even more favorable outcomes (93% local control) for patients undergoing definitive treatment. Multivariate analysis identified vital factors influencing local control, including age, prior surgery, and macrovascular invasion, as well as factors affecting OS, such as gender and prior chemotherapy. This body of research collectively underscores the value of RT, with proton therapy demonstrating significant potential for improving outcomes in challenging liver cancers [201,202].

Furthermore, a pertinent study involving iCCA patients sheds light on the role of liver radiotherapy (L-RT) in advanced liver cancers. While the primary focus was on patients with extrahepatic metastatic disease, the study underscores the potential benefits of RT. Among the findings, iCCA patients with extrahepatic metastatic disease who received L-RT exhibited a median OS of 21 months, a significant improvement compared to patients treated with chemotherapy alone, who had a median OS of 9 months. Notably, treatment-related liver failure was less common among those who received L-RT. These results emphasize the potential advantages of RT, even in the context of challenging CCA cases and in the presence of metastatic disease [203].

## 6. Conclusions

This comprehensive review has shed light on the array of treatment strategies, their outcomes, and potential future directions. Moreover, neoadjuvant chemotherapy, such as durvalumab plus gemcitabine and cisplatin, holds promise for advanced CCA cases, potentially expanding the scope of surgical resection. Interventional therapies, including chemoembolization, have been recognized as valuable approaches for managing unresectable CCA, offering enhanced survival and local disease control. HAIC has demonstrated efficacy in specific cases, while ongoing clinical trials seek to provide deeper insights into these promising approaches. TACE and dbTACE demonstrate localized chemotherapy delivery, fostering tumor regression and prolonged survival. RE, ablative methods such as RFA and IRE, and RT, including proton therapy, offer potential avenues for treating unresectable CCA. Moreover, the expanding realm of targeted therapy in CCA, driven by genetic profiling and gene mutations such as *FGFR2*, *IDH1*, *IDH2*, *NTRK*, *BRAF*, and *HER2*, is revolutionizing personalized treatment approaches. Genetic testing plays a pivotal role in identifying these mutations and guiding treatment choices. CAR-T cell therapy is promising for CCA patients, especially those with specific biomarkers. Despite the progress made, CCA treatment remains intricate, with limited therapeutic options and high mortality rates. This review underscores the necessity for continued research and clinical trials to refine treatment protocols, evaluate emerging therapies, and ultimately enhance the prognosis for CCA patients. Further investigations are essential to establish definitive guidelines for adjuvant, neoadjuvant, and interventional therapies and to explore the potential of immunotherapies. The pursuit of effective CCA treatments is ongoing, and this review contributes to our understanding of this challenging field.

## Figures and Tables

**Table 1:** Age-standardized mortality rate/100 000 population for cholangiocarcinoma (CCA). Intrahepatic cholangiocarcinoma (iCCA), extrahepatic cholangiocarcinoma (eCCA = pCCA + dCCA). Combined mortality of iCCA + eCCA per 100,000. 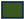 High mortality (>2 cases per 100,000). 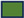 Intermediate mortality (1–2 cases per 100,000). 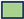 Low mortality (<1 cases per 100 000).

Country	iCCA	eCCA
Republic of Korea	2.6	2.45
Japan	0.97	2.14
Ireland	2.66	0.05
Austria	1.43	0.90
Hong Kong	2.27	0.04
Malta	2.03	0.25
Germany	1.31	0.94
UK	2.04	0.04
Australia	1.96	0.03
Hungary	0.48	1.51
Spain	1.81	0.16
Portugal	1.79	0.12
Canada	1.82	0.07
Switzerland	1.48	0.40
Singapore	1.77	0.10
France	1.79	0.06
Sweden	0.90	0.81
Belgium	1.61	0.09
Netherlands	1.30	0.34
Croatia	0.96	0.57
USA	1.32	0.16
Norway	1.35	0.13
Lithuania	1.04	0.29
Slovakia	0.95	0.37
Czech Republic	0.78	0.52
New Zealand	0.99	0.27
Italy	1.08	0.17
Denmark	1.08	0.14
Latvia	0.96	0.17
Israel	0.95	0.11
Romania	0.55	0.29
Turkey	0.72	0.11
Poland	0.29	0.14

**Table 2:** Summary of the first-line chemotherapeutic options for cholangiocarcinoma (CCA) treatment. * Tegafur, 5-chloro-2,4-dihydropyridine, and potassium oxonate. RCT: randomized controlled trial, OS: overall survival, FU: fluorouracil, LV: leucovorin, PFS: progression-free survival.

First-Line Therapy for CCA				
**Good performance status** **and** **no hyperbilirubinemia**	Regimen	Trial/Evidence	Benefit(s)	Limitation(s)
	**Gemcitabine-based regimens**			
	Gemcitabine plus cisplatin	ABC-02 trial [24] Okusaka et al. RCT [33] Pooled analysis of RCTs [34]	- Leaves open the option of short-term infusional FU plus LV and oxaliplatin (FOLFOX) as a second-line regimen	Higher rates of Grade 3 or 4 neutropenia [35]
	Gemcitabine plus cisplatin and durvalumab	TOPAZ-1 trial [36]	- OS benefit - Longer PFS - Higher objective response rate	Immune-mediated side effects in 13% of patients
	Gemcitabine plus S-1 *	FUGA-BT trial [28]	- Non-inferior to gemcitabine plus cisplatin - Fewer side effects	Limited availability outside the US
	Gemcitabine plus Oxaliplatin (GEMOX)	FUGA-BT trial [28]. Wagner et al. phase III trial [37].	- Better survival outcomes experienced [38]	Results were less favorable in patients who had a poorer performance status or a higher bilirubin level
	GEMOX plus Bevacizumab	Kim et al. phase III RCT [39] Zhu et al. RCT [40]	- Increased PFS - Increased OS	Bevacizumab-related toxicities
	Gemcitabine plus capecitabine	Zhu et al. RCT [40] Nehls et al. RCT [41]	May be an alternative option	Needs RCTs to determine the effectiveness compared to cisplatin
	Gemcitabine plus Nabpaclitaxel	Iqbal et al. RCT [42] Sahai et al. RCT [43]		Neutropenia (43%) and fatigue (14%)
	**Non-gemcitabine-based regimens**			
	Modified FOLFIRINOX (Oxaliplatin, LV, Irinotecan and FU)	Phelip et al. RCT [44] Abdel-Rahman et al. review [45]		No advantages in terms of median OS, PFS, or six-month PFS
**Special Circumstances**	- **Good performance status and hyperbilirubinemia:** 5-FU and LV	Choi et al. trial [46]		
	- **Borderline performance status:**			
	LV-modulated FU	Sanz-Altamira et al. [47] Mayo Clinic regimen [48]		Survival benefits are unclear [47,49,50]
	Capecitabine	Patt et al. [51]	Alternative to LV-modulated FU	Capecitabine as a single agent appears to be relatively less active for CCA than for gallbladder cancer

**Table 3:** Updated data on gene mutations associated with cholangiocarcinoma (CCA).

Year 2020		Year 2022	
Gene Mutations	Percentage	Gene Mutations	Percentage
*FGFR2*	**15–20%**	*FGFR2*	**4–9%**
*IDH1*	**15–20%**	*IDH1*	**3–14%**
*HER2*	**10–15%**	*IDH2*	**4.0%**
*NTRK*	**<5%**	*TMB high*	**3.7%**
*RNF43*	**<5%**	*MDM*	**4.3%**
*MMR*	**<5%**	*BRACA1/2*	**3.4%**
*BRAF*	**<5%**	*ERBB2*	**3.8%**
		*BRAF*	**2.3%**
		*ERBB3*	**1.6%**
		*MSI high*	**1.2%**
		*KRAS*	**1.1%**

**Table 4:** Summary of the targeted therapy for cholangiocarcinoma (CCA) treatment. ORR: overall response, objective response rate, DCR: disease control rate, CR: complete response, PR: partial response, PD: progressive disease, SD: stable disease, MDR: median duration of response, PFS: progression-free survival, HR: hazard ratio, MDS: median duration of survival, BICR: blinded independent central review.

Targeted Therapy for CCA			
Medication	Mechanism	Trial(s)	Results
**FGFR-targeted therapy**			
Derazantinib	ATP-competitive, pan-*FGFR* inhibitor. “*FGFR*1–3 kinases selectivity” [87]	Phase I RCT (NCT01752920) Phase I/II, open-label RCT (NCT01752920)	- ORR (20.7%) - DCR (82.8%)
		Phase II FIDES-01 (NCT03230318)	Ongoing
Erdafitinib	Pan-*FGFR* inhibitor [88]	Phase IIa study (NCT02699606)	- PR 7 (46.7%), SD 5 (33.3%), PD 3 (20.0%) - ORR 7/15 (47%) - DCR12/15 (80%) [89]
Pemigatinib	Selective *FGFR*1–3 inhibitor [90]	FIGHT-202 trial	- DCR 88 (80%) - MDR 7.5 months (95% CI 5.7–14.5) [66] - FDA approval (April 2020) [91]
Infigratinib	Selective inhibitor of *FGFR*1–3 [92]	Phase I study (NCT01004224)	Identification of a recommended phase II dose for infigratinib as 125 mg QD given on a 3-weeks-on/1-week-off schedule [93]
		Phase II study (NCT02150967)	- ORR 23.1% (95% CI: 15.6–32.2%) - MDR 5.0 months (range 0.9–19.1 months) - Median PFS 7.3 months (95% CI: 5.6–7.6 months) [94]
Futibatinib	Pan-*FGFR* inhibitor	Phase I (FOENIX-101; NCT02052778)	- PR 5 (5.8%; three patients with FGFR2 fusion-positive iCCA, and two patients with FGFR1-mutated primary brain tumor) [95]
		FOENIX-CCA2 phase II trial (NCT02052778)	- ORR (37.3%) - DCR (82.1%) [96]
**IDH- targeted therapy**			
Ivosidenib	*IDH1* inhibitor	Combined phase I/II study	- Median PFS 2.7 months [95% CI 1.6–4.2] vs. 1.4 months [1.4–1.6] - HR (0.37; 95% CI 0.25–0.54; one-sided *p* < 0·0001) [63] - FDA approval (August 2021) [97]
**TRK fusion- targeted therapy**			
Larotrectinib	Tropomyosin receptor kinase (TRK) inhibitor	Analysis of (NCT02122913 and NCT02576431) trials	- ORR (76%) (CR 9%) - Confirmed PR (57%), PR pending confirmation (9%) SD (12%), PD (11%) [98] - FDA approval (November 2018) [99]
		Analysis of (NCT02122913, NCT02637687, and NCT02576431) trials	- Larotrectinib was well-tolerated and induced fast, lasting responses in TRK fusion GI cancers. (2 CCA patients) [100]
Entrectinib	Inhibitor of *NTRK1/2/3*, *ROS1*, and *ALK* [101]	An updated integrated analysis phase I/II studies (ALKA, STARTRK-1, STARTRK-2; EudraCT 2012-000148-88; NCT02097810; NCT02568267)	- MDS follow-up 14.2 mo (range 0.1–29.7) - BICR ORR 63.5% (95% CI 51.5–74.4), CRs 5 (6.8%) - Median BICR DOR 12.9 mo (95% CI 9.3–NE) - Median BICR PFS 11.2 mo (95% CI 8.0–15.7) - Median OS 23.9 mo (16.0–NE) [102]
**BRAF V600E- targeted therapy**			
Combination of dabrafenib plus trametinib	Reversibly and selectively inhibits mitogen-activated extracellular kinase *[MEK]*, a downstream effector of *BRAF*	Phase II (ROAR) basket trial	- OR 22 (51%, 95% CI 36–67) of 43 patients [103]
		NCI-MATCH Trial Subprotocol H	- Confirmed ORR 38% (90% CI, 22.9–54.9%, *p* < 0.0001) - PFS 11.4 months (90% CI, 8.4 to 16.3 months) - The median OS was 28.6 months [69] - FDA approval (June 2022) [104]
**HER2 overexpression- targeted therapy**			
Combination of pertuzumab plus trastuzumab		MyPathway HER2 basket study	- Objective antitumor response in 9 of 39 HER2 amplified/overexpressed RAS wild-type biliary tract cancers (23%) - MDR 10.8 months [105]
**EGFR-targeted therapy**			
Erlotinib	Oral tyrosine kinase inhibitor	Phase II RCT	- Progression free in 7 patients (17%; 95% CI, 7–31%) at 6 months [106]

## References

[1] Chun Y.S., Pawlik T.M., Vauthey J.N. (2018). 8th Edition of the AJCC Cancer Staging Manual: Pancreas and Hepatobiliary Cancers. Ann. Surg. Oncol..

[2] Blechacz B., Komuta M., Roskams T., Gores G.J. (2011). Clinical diagnosis and staging of cholangiocarcinoma. Nat. Rev. Gastroenterol. Hepatol..

[3] Bismuth H., Nakache R., Diamond T. (1992). Management strategies in resection for hilar cholangiocarcinoma. Ann. Surg..

[4] Banales J.M., Marin J.J.G., Lamarca A., Rodrigues P.M., Khan S.A., Roberts L.R., Cardinale V., Carpino G., Andersen J.B., Braconi C. (2020). Cholangiocarcinoma 2020: The next horizon in mechanisms and management. Nat. Rev. Gastroenterol. Hepatol..

[5] Banales J.M., Cardinale V., Carpino G., Marzioni M., Andersen J.B., Invernizzi P., Lind G.E., Folseraas T., Forbes S.J., Fouassier L. (2016). Expert consensus document: Cholangiocarcinoma: Current knowledge and future perspectives consensus statement from the European Network for the Study of Cholangiocarcinoma (ENS-CCA). Nat. Rev. Gastroenterol. Hepatol..

[6] Sripa B., Kaewkes S., Sithithaworn P., Mairiang E., Laha T., Smout M., Pairojkul C., Bhudhisawasdi V., Tesana S., Thinkamrop B. (2007). Liver fluke induces cholangiocarcinoma. PLoS Med..

[7] Shaib Y.H., Davila J.A., McGlynn K., El-Serag H.B. (2004). Rising incidence of intrahepatic cholangiocarcinoma in the United States: A true increase?. J. Hepatol..

[8] West J., Wood H., Logan R.F., Quinn M., Aithal G.P. (2006). Trends in the incidence of primary liver and biliary tract cancers in England and Wales 1971–2001. Br. J. Cancer.

[9] Patel T. (2001). Increasing incidence and mortality of primary intrahepatic cholangiocarcinoma in the United States. Hepatology.

[10] Patel N., Benipal B. (2019). Incidence of Cholangiocarcinoma in the USA from 2001 to 2015: A US Cancer Statistics Analysis of 50 States. Cureus.

[11] Shaib Y.H., El-Serag H.B., Davila J.A., Morgan R., McGlynn K.A. (2005). Risk factors of intrahepatic cholangiocarcinoma in the United States: A case-control study. Gastroenterology.

[12] Broomé U., Olsson R., Lööf L., Bodemar G., Hultcrantz R., Danielsson A., Prytz H., Sandberg-Gertzén H., Wallerstedt S., Lindberg G. (1996). Natural history and prognostic factors in 305 Swedish patients with primary sclerosing cholangitis. Gut.

[13] Chen M.F. (1999). Peripheral cholangiocarcinoma (cholangiocellular carcinoma): Clinical features, diagnosis and treatment. J. Gastroenterol. Hepatol..

[14] Shin H.-R., Lee C.-U., Park H.-J., Seol S.-Y., Chung J.-M., Choi H.-C., Ahn Y.-O., Shigemastu T. (1996). Hepatitis B and C Virus, Clonorchis sinensis for the Risk of Liver Cancer: A Case-Control Study in Pusan, Korea. Int. J. Epidemiol..

[15] De Lorenzo S., Tovoli F., Mazzotta A., Vasuri F., Edeline J., Malvi D., Boudjema K., Renzulli M., Jeddou H., D’Errico A. (2020). Non-Alcoholic Steatohepatitis as a Risk Factor for Intrahepatic Cholangiocarcinoma and Its Prognostic Role. Cancers.

[16] Yi M., Peng W., Feng X., Teng F., Tang Y., Kong Q., Chen Z. (2022). Extrahepatic morbidities and mortality of NAFLD: An umbrella review of meta-analyses. Aliment. Pharmacol. Ther..

[17] Howlader N., Noone A.M., Krapcho M., Miller D., Bishop K., Kosary C.L., Yu M., Ruhl J., Tatalovich Z., Mariotto A. (2016). SEER Cancer Statistics Review, 1975–2014.

[18] Mihalache F., Tantau M., Diaconu B., Acalovschi M. (2010). Survival and quality of life of cholangiocarcinoma patients: A prospective study over a 4 year period. J. Gastrointest. Liver Dis. JGLD.

[19] Abboud K., Umoru G., Esmail A., Abudayyeh A., Murakami N., Al-Shamsi H.O., Javle M., Saharia A., Connor A.A., Kodali S. (2023). Immune checkpoint inhibitors for solid tumors in the adjuvant setting: Current progress, future directions, and role in transplant oncology. Cancers.

[20] Cho S.M., Esmail A., Raza A., Dacha S., Abdelrahim M. (2022). Timeline of FDA-approved targeted therapy for cholangiocarcinoma. Cancers.

[21] Zhang Y., Esmail A., Mazzaferro V., Abdelrahim M. (2022). Newest Therapies for Cholangiocarcinoma: An Updated Overview of Approved Treatments with Transplant Oncology Vision. Cancers.

[22] Pignon J.P., Tribodet H., Scagliotti G.V., Douillard J.Y., Shepherd F.A., Stephens R.J., Dunant A., Torri V., Rosell R., Seymour L. (2008). Lung adjuvant cisplatin evaluation: A pooled analysis by the LACE Collaborative Group. J. Clin. Oncol..

[23] Tisnado D., Malin J., Kahn K., Landrum M.B., Fletcher R., Klabunde C., Clauser S., Rogers S.O., Keating N.L. (2016). Variations in Oncologist Recommendations for Chemotherapy for Stage IV Lung Cancer: What Is the Role of Performance Status?. J. Oncol. Pract..

[24] Valle J., Wasan H., Palmer D.H., Cunningham D., Anthoney A., Maraveyas A., Madhusudan S., Iveson T., Hughes S., Pereira S.P. (2010). Cisplatin plus gemcitabine versus gemcitabine for biliary tract cancer. N. Engl. J. Med..

[25] Furuse J., Okusaka T., Boku N., Ohkawa S., Sawaki A., Masumoto T., Funakoshi A. (2008). S-1 monotherapy as first-line treatment in patients with advanced biliary tract cancer: A multicenter phase II study. Cancer Chemother. Pharmacol..

[26] Kanai M., Yoshimura K., Tsumura T., Asada M., Suzuki C., Niimi M., Matsumoto S., Nishimura T., Nitta T., Yasuchika K. (2011). A multi-institution phase II study of gemcitabine/S-1 combination chemotherapy for patients with advanced biliary tract cancer. Cancer Chemother. Pharmacol..

[27] Sasaki T., Isayama H., Nakai Y., Ito Y., Kogure H., Togawa O., Toda N., Yasuda I., Hasebe O., Maetani I. (2010). Multicenter, phase II study of gemcitabine and S-1 combination chemotherapy in patients with advanced biliary tract cancer. Cancer Chemother. Pharmacol..

[28] Morizane C., Okusaka T., Mizusawa J., Katayama H., Ueno M., Ikeda M., Ozaka M., Okano N., Sugimori K., Fukutomi A. (2019). Combination gemcitabine plus S-1 versus gemcitabine plus cisplatin for advanced/recurrent biliary tract cancer: The FUGA-BT (JCOG1113) randomized phase III clinical trial. Ann. Oncol..

[29] Ioka T., Kanai M., Kobayashi S., Sakai D., Eguchi H., Baba H., Seo S., Taketomi A., Takayama T., Yamaue H. (2023). Randomized phase III study of gemcitabine, cisplatin plus S-1 versus gemcitabine, cisplatin for advanced biliary tract cancer (KHBO1401-MITSUBA). J. Hepatobiliary Pancreat. Sci..

[30] Lamarca A., Palmer D.H., Wasan H.S., Ross P.J., Ma Y.T., Arora A., Falk S., Gillmore R., Wadsley J., Patel K. (2021). Second-line FOLFOX chemotherapy versus active symptom control for advanced biliary tract cancer (ABC-06): A phase 3, open-label, randomised, controlled trial. Lancet Oncol..

[31] Glimelius B., Hoffman K., Sjödén P.O., Jacobsson G., Sellström H., Enander L.K., Linné T., Svensson C. (1996). Chemotherapy improves survival and quality of life in advanced pancreatic and biliary cancer. Ann. Oncol..

[32] Oh D.-Y., He A.R., Qin S., Chen L.-T., Okusaka T., Vogel A., Kim J.W., Suksombooncharoen T., Lee M.A., Kitano M. (2022). Durvalumab plus Gemcitabine and Cisplatin in Advanced Biliary Tract Cancer. NEJM Evid..

[33] Okusaka T., Nakachi K., Fukutomi A., Mizuno N., Ohkawa S., Funakoshi A., Nagino M., Kondo S., Nagaoka S., Funai J. (2010). Gemcitabine alone or in combination with cisplatin in patients with biliary tract cancer: A comparative multicentre study in Japan. Br. J. Cancer.

[34] Eckel F., Schmid R.M. (2007). Chemotherapy in advanced biliary tract carcinoma: A pooled analysis of clinical trials. Br. J. Cancer.

[35] Bridgewater J., Lopes A., Palmer D., Cunningham D., Anthoney A., Maraveyas A., Madhusudan S., Iveson T., Valle J., Wasan H. (2016). Quality of life, long-term survivors and long-term outcome from the ABC-02 study. Br. J. Cancer.

[36] Oh D.Y., Lee K.H., Lee D.W., Yoon J., Kim T.Y., Bang J.H., Nam A.R., Oh K.S., Kim J.M., Lee Y. (2022). Gemcitabine and cisplatin plus durvalumab with or without tremelimumab in chemotherapy-naive patients with advanced biliary tract cancer: An open-label, single-centre, phase 2 study. Lancet Gastroenterol. Hepatol..

[37] Wagner A.D., Buechner-Steudel P., Moehler M., Schmalenberg H., Behrens R., Fahlke J., Wein A., Behl S., Kuss O., Kleber G. (2009). Gemcitabine, oxaliplatin and 5-FU in advanced bile duct and gallbladder carcinoma: Two parallel, multicentre phase-II trials. Br. J. Cancer.

[38] Yang R., Wang B., Chen Y.J., Li H.B., Hu J.B., Zou S.Q. (2013). Efficacy of gemcitabine plus platinum agents for biliary tract cancers: A meta-analysis. Anti-Cancer Drugs.

[39] Kim S.T., Kang J.H., Lee J., Lee H.W., Oh S.Y., Jang J.S., Lee M.A., Sohn B.S., Yoon S.Y., Choi H.J. (2019). Capecitabine plus oxaliplatin versus gemcitabine plus oxaliplatin as first-line therapy for advanced biliary tract cancers: A multicenter, open-label, randomized, phase III, noninferiority trial. Ann. Oncol..

[40] Zhu A.X., Meyerhardt J.A., Blaszkowsky L.S., Kambadakone A.R., Muzikansky A., Zheng H., Clark J.W., Abrams T.A., Chan J.A., Enzinger P.C. (2010). Efficacy and safety of gemcitabine, oxaliplatin, and bevacizumab in advanced biliary-tract cancers and correlation of changes in 18-fluorodeoxyglucose PET with clinical outcome: A phase 2 study. Lancet Oncol..

[41] Nehls O., Oettle H., Hartmann J.T., Hofheinz R.D., Hass H.G., Horger M.S., Koppenhöfer U., Hochhaus A., Stieler J., Trojan J. (2008). Capecitabine plus oxaliplatin as first-line treatment in patients with advanced biliary system adenocarcinoma: A prospective multicentre phase II trial. Br. J. Cancer.

[42] Iqbal S., Rankin C., Lenz H.J., Gold P.J., Ahmad S.A., El-Khoueiry A.B., Messino M.J., Holcombe R.F., Blanke C.D. (2011). A phase II trial of gemcitabine and capecitabine in patients with unresectable or metastatic gallbladder cancer or cholangiocarcinoma: Southwest Oncology Group study S0202. Cancer Chemother. Pharmacol..

[43] Sahai V., Catalano P.J., Zalupski M.M., Lubner S.J., Menge M.R., Nimeiri H.S., Munshi H.G., Benson A.B., O’Dwyer P.J. (2018). Nab-Paclitaxel and Gemcitabine as First-line Treatment of Advanced or Metastatic Cholangiocarcinoma: A Phase 2 Clinical Trial. JAMA Oncol..

[44] Phelip J.M., Desrame J., Edeline J., Barbier E., Terrebonne E., Michel P., Perrier H., Dahan L., Bourgeois V., Akouz F.K. (2022). Modified FOLFIRINOX Versus CISGEM Chemotherapy for Patients With Advanced Biliary Tract Cancer (PRODIGE 38 AMEBICA): A Randomized Phase II Study. J. Clin. Oncol..

[45] Abdel-Rahman O., Elsayed Z., Elhalawani H. (2018). Gemcitabine-based chemotherapy for advanced biliary tract carcinomas. Cochrane Database Syst. Rev..

[46] Choi C.W., Choi I.K., Seo J.H., Kim B.S., Kim J.S., Kim C.D., Um S.H., Kim J.S., Kim Y.H. (2000). Effects of 5-fluorouracil and leucovorin in the treatment of pancreatic-biliary tract adenocarcinomas. Am. J. Clin. Oncol..

[47] Sanz-Altamira P.M., Ferrante K., Jenkins R.L., Lewis W.D., Huberman M.S., Stuart K.E. (1998). A phase II trial of 5-fluorouracil, leucovorin, and carboplatin in patients with unresectable biliary tree carcinoma. Cancer.

[48] Malik I.A., Aziz Z. (2003). Prospective evaluation of efficacy and toxicity of 5-fu and folinic acid (Mayo Clinic regimen) in patients with advanced cancer of the gallbladder. Am. J. Clin. Oncol..

[49] Takada T., Kato H., Matsushiro T., Nimura Y., Nagakawa T., Nakayama T. (1994). Comparison of 5-fluorouracil, doxorubicin and mitomycin C with 5-fluorouracil alone in the treatment of pancreatic-biliary carcinomas. Oncology.

[50] Ducreux M., Rougier P., Fandi A., Clavero-Fabri M.C., Villing A.L., Fassone F., Fandi L., Zarba J., Armand J.P. (1998). Effective treatment of advanced biliary tract carcinoma using 5-fluorouracil continuous infusion with cisplatin. Ann. Oncol..

[51] Patt Y.Z., Hassan M.M., Aguayo A., Nooka A.K., Lozano R.D., Curley S.A., Vauthey J.N., Ellis L.M., Schnirer I.I., Wolff R.A. (2004). Oral capecitabine for the treatment of hepatocellular carcinoma, cholangiocarcinoma, and gallbladder carcinoma. Cancer.

[52] Lamarca A., Kapacee Z., Breeze M., Bell C., Belcher D., Staiger H., Taylor C., McNamara M.G., Hubner R.A., Valle J.W. (2020). Molecular Profiling in Daily Clinical Practice: Practicalities in Advanced Cholangiocarcinoma and Other Biliary Tract Cancers. J. Clin. Med..

[53] Sia D., Hoshida Y., Villanueva A., Roayaie S., Ferrer J., Tabak B., Peix J., Sole M., Tovar V., Alsinet C. (2013). Integrative molecular analysis of intrahepatic cholangiocarcinoma reveals 2 classes that have different outcomes. Gastroenterology.

[54] Rizvi S., Borad M.J. (2016). The rise of the FGFR inhibitor in advanced biliary cancer: The next cover of time magazine?. J. Gastrointest. Oncol..

[55] Walter D., Hartmann S., Waidmann O. (2017). Update on cholangiocarcinoma: Potential impact of genomic studies on clinical management. Z. Gastroenterol..

[56] Yoshikawa D., Ojima H., Iwasaki M., Hiraoka N., Kosuge T., Kasai S., Hirohashi S., Shibata T. (2008). Clinicopathological and prognostic significance of EGFR, VEGF, and HER2 expression in cholangiocarcinoma. Br. J. Cancer.

[57] Oliveira D.V., Zhang S., Chen X., Calvisi D.F., Andersen J.B. (2017). Molecular profiling of intrahepatic cholangiocarcinoma: The search for new therapeutic targets. Expert. Rev. Gastroenterol. Hepatol..

[58] Bahleda R., Italiano A., Hierro C., Mita A., Cervantes A., Chan N., Awad M., Calvo E., Moreno V., Govindan R. (2019). Multicenter Phase I Study of Erdafitinib (JNJ-42756493), Oral Pan-Fibroblast Growth Factor Receptor Inhibitor, in Patients with Advanced or Refractory Solid Tumors. Clin. Cancer Res..

[59] Klein O., Kee D., Nagrial A., Markman B., Underhill C., Michael M., Jackett L., Lum C., Behren A., Palmer J. (2020). Evaluation of Combination Nivolumab and Ipilimumab Immunotherapy in Patients With Advanced Biliary Tract Cancers: Subgroup Analysis of a Phase 2 Nonrandomized Clinical Trial. JAMA Oncol..

[60] Ross J.S., Wang K., Gay L., Al-Rohil R., Rand J.V., Jones D.M., Lee H.J., Sheehan C.E., Otto G.A., Palmer G. (2014). New routes to targeted therapy of intrahepatic cholangiocarcinomas revealed by next-generation sequencing. Oncologist.

[61] Farshidfar F., Zheng S., Gingras M.C., Newton Y., Shih J., Robertson A.G., Hinoue T., Hoadley K.A., Gibb E.A., Roszik J. (2017). Integrative Genomic Analysis of Cholangiocarcinoma Identifies Distinct IDH-Mutant Molecular Profiles. Cell Rep..

[62] Chakravarty D., Johnson A., Sklar J., Lindeman N.I., Moore K., Ganesan S., Lovly C.M., Perlmutter J., Gray S.W., Hwang J. (2022). Somatic Genomic Testing in Patients With Metastatic or Advanced Cancer: ASCO Provisional Clinical Opinion. J. Clin. Oncol..

[63] Abou-Alfa G.K., Macarulla T., Javle M.M., Kelley R.K., Lubner S.J., Adeva J., Cleary J.M., Catenacci D.V., Borad M.J., Bridgewater J. (2020). Ivosidenib in IDH1-mutant, chemotherapy-refractory cholangiocarcinoma (ClarIDHy): A multicentre, randomised, double-blind, placebo-controlled, phase 3 study. Lancet Oncol..

[64] Mazzaferro V., El-Rayes B.F., Droz Dit Busset M., Cotsoglou C., Harris W.P., Damjanov N., Masi G., Rimassa L., Personeni N., Braiteh F. (2019). Derazantinib (ARQ 087) in advanced or inoperable FGFR2 gene fusion-positive intrahepatic cholangiocarcinoma. Br. J. Cancer.

[65] Mody K., Jain P., El-Refai S.M., Azad N.S., Zabransky D.J., Baretti M., Shroff R.T., Kelley R.K., El-Khouiery A.B., Hockenberry A.J. (2022). Clinical, Genomic, and Transcriptomic Data Profiling of Biliary Tract Cancer Reveals Subtype-Specific Immune Signatures. JCO Precis. Oncol..

[66] Abou-Alfa G.K., Sahai V., Hollebecque A., Vaccaro G., Melisi D., Al-Rajabi R., Paulson A.S., Borad M.J., Gallinson D., Murphy A.G. (2020). Pemigatinib for previously treated, locally advanced or metastatic cholangiocarcinoma: A multicentre, open-label, phase 2 study. Lancet Oncol..

[67] Lavacchi D., Caliman E., Rossi G., Buttitta E., Botteri C., Fancelli S., Pellegrini E., Roviello G., Pillozzi S., Antonuzzo L. (2022). Ivosidenib in IDH1-mutated cholangiocarcinoma: Clinical evaluation and future directions. Pharmacol. Ther..

[68] ten Haaft B.H.E.A., Pedregal M., Prato J., Klümpen H.-J., Moreno V., Lamarca A. (2024). Revolutionizing anti-HER2 therapies for extrahepatic cholangiocarcinoma and gallbladder cancer: Current advancements and future perspectives. Eur. J. Cancer.

[69] Salama A.K.S., Li S., Macrae E.R., Park J.I., Mitchell E.P., Zwiebel J.A., Chen H.X., Gray R.J., McShane L.M., Rubinstein L.V. (2020). Dabrafenib and Trametinib in Patients With Tumors With BRAF(V600E) Mutations: Results of the NCI-MATCH Trial Subprotocol H. J. Clin. Oncol..

[70] Wen P.Y., Stein A., van den Bent M., De Greve J., Wick A., de Vos F., von Bubnoff N., van Linde M.E., Lai A., Prager G.W. (2022). Dabrafenib plus trametinib in patients with BRAF(V600E)-mutant low-grade and high-grade glioma (ROAR): A multicentre, open-label, single-arm, phase 2, basket trial. Lancet Oncol..

[71] Nam A.R., Kim J.W., Cha Y., Ha H., Park J.E., Bang J.H., Jin M.H., Lee K.H., Kim T.Y., Han S.W. (2016). Therapeutic implication of HER2 in advanced biliary tract cancer. Oncotarget.

[72] Jusakul A., Cutcutache I., Yong C.H., Lim J.Q., Huang M.N., Padmanabhan N., Nellore V., Kongpetch S., Ng A.W.T., Ng L.M. (2017). Whole-Genome and Epigenomic Landscapes of Etiologically Distinct Subtypes of Cholangiocarcinoma. Cancer Discov..

[73] Javle M., Bekaii-Saab T., Jain A., Wang Y., Kelley R.K., Wang K., Kang H.C., Catenacci D., Ali S., Krishnan S. (2016). Biliary cancer: Utility of next-generation sequencing for clinical management. Cancer.

[74] Patel T.H., Marcus L., Horiba M.N., Donoghue M., Chatterjee S., Mishra-Kalyani P.S., Schuck R.N., Li Y., Zhang X., Fourie Zirkelbach J. (2023). FDA Approval Summary: Pemigatinib for Previously Treated, Unresectable Locally Advanced or Metastatic Cholangiocarcinoma with FGFR2 Fusion or Other Rearrangement. Clin. Cancer Res..

[75] Gandhy S.U., Casak S.J., Mushti S.L., Cheng J., Subramaniam S., Zhao H., Zhao M., Bi Y., Liu G., Fan J. (2023). FDA Approval Summary: Futibatinib for Unresectable Advanced or Metastatic, Chemotherapy Refractory Intrahepatic Cholangiocarcinoma with FGFR2 Fusions or Other Rearrangements. Clin. Cancer Res..

[76] Storandt M.H., Kurniali P.C., Mahipal A., Jin Z. (2023). Targeted Therapies in Advanced Cholangiocarcinoma. Life.

[77] Kwok G., Yau T.C., Chiu J.W., Tse E., Kwong Y.L. (2016). Pembrolizumab (Keytruda). Hum. Vaccines Immunother..

[78] Sasaki T., Isayama H., Nakai Y., Ito Y., Yasuda I., Toda N., Kogure H., Hanada K., Maguchi H., Sasahira N. (2013). A randomized phase II study of gemcitabine and S-1 combination therapy versus gemcitabine monotherapy for advanced biliary tract cancer. Cancer Chemother. Pharmacol..

[79] Neuzillet C., Casadei Gardini A., Brieau B., Vivaldi C., Smolenschi C., Brandi G., Tougeron D., Filippi R., Vienot A., Silvestris N. (2019). Prediction of survival with second-line therapy in biliary tract cancer: Actualisation of the AGEO CT2BIL cohort and European multicentre validations. Eur. J. Cancer.

[80] He S., Shen J., Sun X., Liu L., Dong J. (2014). A phase II FOLFOX-4 regimen as second-line treatment in advanced biliary tract cancer refractory to gemcitabine/cisplatin. J. Chemother..

[81] Zhang D., Dorman K., Westphalen C.B., Haas M., Ormanns S., Neumann J., Seidensticker M., Ricke J., De Toni E.N., Klauschen F. (2024). Unresectable biliary tract cancer: Current and future systemic therapy. Eur. J. Cancer.

[82] Kim B.J., Yoo C., Kim K.P., Hyung J., Park S.J., Ryoo B.Y., Chang H.M. (2017). Efficacy of fluoropyrimidine-based chemotherapy in patients with advanced biliary tract cancer after failure of gemcitabine plus cisplatin: Retrospective analysis of 321 patients. Br. J. Cancer.

[83] Guion-Dusserre J.F., Lorgis V., Vincent J., Bengrine L., Ghiringhelli F. (2015). FOLFIRI plus bevacizumab as a second-line therapy for metastatic intrahepatic cholangiocarcinoma. World J. Gastroenterol..

[84] Lubner S.J., Mahoney M.R., Kolesar J.L., Loconte N.K., Kim G.P., Pitot H.C., Philip P.A., Picus J., Yong W.P., Horvath L. (2010). Report of a multicenter phase II trial testing a combination of biweekly bevacizumab and daily erlotinib in patients with unresectable biliary cancer: A phase II Consortium study. J. Clin. Oncol..

[85] Bréchon M., Dior M., Dréanic J., Brieau B., Guillaumot M.A., Brezault C., Mir O., Goldwasser F., Coriat R. (2018). Addition of an antiangiogenic therapy, bevacizumab, to gemcitabine plus oxaliplatin improves survival in advanced biliary tract cancers. Investig. New Drugs.

[86] Demols A., Borbath I., Van den Eynde M., Houbiers G., Peeters M., Marechal R., Delaunoit T., Goemine J.C., Laurent S., Holbrechts S. (2020). Regorafenib after failure of gemcitabine and platinum-based chemotherapy for locally advanced/metastatic biliary tumors: REACHIN, a randomized, double-blind, phase II trial. Ann. Oncol..

[87] Hall T.G., Yu Y., Eathiraj S., Wang Y., Savage R.E., Lapierre J.M., Schwartz B., Abbadessa G. (2016). Preclinical Activity of ARQ 087, a Novel Inhibitor Targeting FGFR Dysregulation. PLoS ONE.

[88] Perera T.P.S., Jovcheva E., Mevellec L., Vialard J., De Lange D., Verhulst T., Paulussen C., Van De Ven K., King P., Freyne E. (2017). Discovery and Pharmacological Characterization of JNJ-42756493 (Erdafitinib), a Functionally Selective Small-Molecule FGFR Family Inhibitor. Mol. Cancer Ther..

[89] Park J.O., Feng Y.-H., Chen Y.-Y., Su W.-C., Oh D.-Y., Shen L., Kim K.-P., Liu X., Bai Y., Liao H. (2019). Updated results of a phase IIa study to evaluate the clinical efficacy and safety of erdafitinib in Asian advanced cholangiocarcinoma (CCA) patients with FGFR alterations. J. Clin. Oncol..

[90] Liu P.C.C., Koblish H., Wu L., Bowman K., Diamond S., DiMatteo D., Zhang Y., Hansbury M., Rupar M., Wen X. (2020). INCB054828 (pemigatinib), a potent and selective inhibitor of fibroblast growth factor receptors 1, 2, and 3, displays activity against genetically defined tumor models. PLoS ONE.

[91] Food and Drug Administration FDA Grants Accelerated Approval to Pemigatinib for Cholangiocarcinoma with an FGFR2 Rearrangement or Fusion. www.fda.gov/drugs/resources-information-approved-drugs/fda-grants-accelerated-approval-pemigatinib-cholangiocarcinoma-fgfr2-rearrangement-or-fusion.

[92] Guagnano V., Furet P., Spanka C., Bordas V., Le Douget M., Stamm C., Brueggen J., Jensen M.R., Schnell C., Schmid H. (2011). Discovery of 3-(2,6-dichloro-3,5-dimethoxy-phenyl)-1-{6-[4-(4-ethyl-piperazin-1-yl)-phenylamino]-pyrimidin-4-yl}-1-methyl-urea (NVP-BGJ398), a potent and selective inhibitor of the fibroblast growth factor receptor family of receptor tyrosine kinase. J. Med. Chem..

[93] Nogova L., Sequist L.V., Perez Garcia J.M., Andre F., Delord J.P., Hidalgo M., Schellens J.H., Cassier P.A., Camidge D.R., Schuler M. (2017). Evaluation of BGJ398, a Fibroblast Growth Factor Receptor 1–3 Kinase Inhibitor, in Patients With Advanced Solid Tumors Harboring Genetic Alterations in Fibroblast Growth Factor Receptors: Results of a Global Phase I, Dose-Escalation and Dose-Expansion Study. J. Clin. Oncol..

[94] Javle M.M., Roychowdhury S., Kelley R.K., Sadeghi S., Macarulla T., Waldschmidt D.T., Goyal L., Borbath I., El-Khoueiry A.B., Yong W.-P. (2021). Final results from a phase II study of infigratinib (BGJ398), an FGFR-selective tyrosine kinase inhibitor, in patients with previously treated advanced cholangiocarcinoma harboring an FGFR2 gene fusion or rearrangement. J. Clin. Oncol..

[95] Sootome H., Fujita H., Ito K., Ochiiwa H., Fujioka Y., Ito K., Miura A., Sagara T., Ito S., Ohsawa H. (2020). Futibatinib Is a Novel Irreversible FGFR 1-4 Inhibitor That Shows Selective Antitumor Activity against FGFR-Deregulated Tumors. Cancer Res..

[96] Maggie L.S. (2022). FOENIX Update: A New Era in Cholangiocarcinoma Precision Medicine. Evid. -Based Oncol..

[97] Zhu A.X., Macarulla T., Javle M.M., Kelley R.K., Lubner S.J., Adeva J., Cleary J.M., Catenacci D.V.T., Borad M.J., Bridgewater J.A. (2021). Final Overall Survival Efficacy Results of Ivosidenib for Patients With Advanced Cholangiocarcinoma With IDH1 Mutation: The Phase 3 Randomized Clinical ClarIDHy Trial. JAMA Oncol..

[98] Berlin J., Hong D.S., Deeken J.F., Boni V., Oh D.-Y., Patel J.D., Nanda S., Brega N., Childs B.H., Hyman D.M. (2020). Efficacy and safety of larotrectinib in patients with TRK fusion gastrointestinal cancer. J. Clin. Oncol..

[99] Food and Drug Administration (2018). FDA Approves Larotrectinib for Solid Tumors with NTRK Gene Fusions.

[100] Nathenson M., Demetri G., Lassen U., Hong D., Boni V., Deeken J., Dowlati A., Cox M., Ku N., Cruickshank S. (2018). Activity of larotrectinib in patients with TRK fusion GI malignancies. Ann. Oncol..

[101] Drilon A., Siena S., Ou S.I., Patel M., Ahn M.J., Lee J., Bauer T.M., Farago A.F., Wheler J.J., Liu S.V. (2017). Safety and Antitumor Activity of the Multitargeted Pan-TRK, ROS1, and ALK Inhibitor Entrectinib: Combined Results from Two Phase I Trials (ALKA-372-001 and STARTRK-1). Cancer Discov..

[102] Rolfo C.D., Braud F.G.D., Doebele R.C., Drilon A.E., Siena S., Patel M., Cho B.C., Liu S.V., Ahn M.-J., Chiu C.-H. (2020). Efficacy and safety of entrectinib in patients (pts) with NTRK-fusion positive (NTRK-fp) solid tumors: An updated integrated analysis. J. Clin. Oncol..

[103] Subbiah V., Lassen U., Élez E., Italiano A., Curigliano G., Javle M., de Braud F., Prager G.W., Greil R., Stein A. (2020). Dabrafenib plus trametinib in patients with BRAF(V600E)-mutated biliary tract cancer (ROAR): A phase 2, open-label, single-arm, multicentre basket trial. Lancet Oncol..

[104] Center for Drug Evaluation and Research (2022). FDA Grants Accelerated Approval to Dabrafenib in Combination with Trametinib for Unresectable or Metastatic Solid Tumors with BRAF V600E Mutation.

[105] Meric-Bernstam F., Hainsworth J., Bose R., Howard A.B., Friedman C.F., Kurzrock R., Swanton C., Wang Y., Levy J., Schulze K. (2021). MyPathway HER2 basket study: Pertuzumab (P) + trastuzumab (H) treatment of a large, tissue-agnostic cohort of patients with HER2-positive advanced solid tumors. J. Clin. Oncol..

[106] Philip P.A., Mahoney M.R., Allmer C., Thomas J., Pitot H.C., Kim G., Donehower R.C., Fitch T., Picus J., Erlichman C. (2006). Phase II study of erlotinib in patients with advanced biliary cancer. J. Clin. Oncol..

[107] Sato Y., Kinoshita M., Takemura S., Tanaka S., Hamano G., Nakamori S., Fujikawa M., Sugawara Y., Yamamoto T., Arimoto A. (2017). The PD-1/PD-L1 axis may be aberrantly activated in occupational cholangiocarcinoma. Pathol. Int..

[108] Im S.J., Hashimoto M., Gerner M.Y., Lee J., Kissick H.T., Burger M.C., Shan Q., Hale J.S., Lee J., Nasti T.H. (2016). Defining CD8+ T cells that provide the proliferative burst after PD-1 therapy. Nature.

[109] Piha-Paul S.A., Oh D.-Y., Ueno M., Malka D., Chung H.C., Nagrial A., Kelley R.K., Ros W., Italiano A., Nakagawa K. (2020). Efficacy and safety of pembrolizumab for the treatment of advanced biliary cancer: Results from the KEYNOTE-158 and KEYNOTE-028 studies. Int. J. Cancer.

[110] Kim R.D., Chung V., Alese O.B., El-Rayes B.F., Li D., Al-Toubah T.E., Schell M.J., Zhou J.M., Mahipal A., Kim B.H. (2020). A Phase 2 Multi-institutional Study of Nivolumab for Patients With Advanced Refractory Biliary Tract Cancer. JAMA Oncol..

[111] El-Khoueiry A.B., Sangro B., Yau T., Crocenzi T.S., Kudo M., Hsu C., Kim T.Y., Choo S.P., Trojan J., Welling T.H.R. (2017). Nivolumab in patients with advanced hepatocellular carcinoma (CheckMate 040): An open-label, non-comparative, phase 1/2 dose escalation and expansion trial. Lancet.

[112] Zhang J., Endres S., Kobold S. (2019). Enhancing tumor T cell infiltration to enable cancer immunotherapy. Immunotherapy.

[113] Guo Y., Feng K., Liu Y., Wu Z., Dai H., Yang Q., Wang Y., Jia H., Han W. (2018). Phase I Study of Chimeric Antigen Receptor-Modified T Cells in Patients with EGFR-Positive Advanced Biliary Tract Cancers. Clin. Cancer Res..

[114] Yu L., Wang J. (2019). T cell-redirecting bispecific antibodies in cancer immunotherapy: Recent advances. J. Cancer Res. Clin. Oncol..

[115] Thakur A., Scholler J., Schalk D.L., June C.H., Lum L.G. (2020). Enhanced cytotoxicity against solid tumors by bispecific antibody-armed CD19 CAR T cells: A proof-of-concept study. J. Cancer Res. Clin. Oncol..

[116] Hamid O., Ismail R., Puzanov I. (2020). Intratumoral Immunotherapy-Update 2019. Oncologist.

[117] Lange S., Lampe J., Bossow S., Zimmermann M., Neubert W., Bitzer M., Lauer U.M. (2013). A novel armed oncolytic measles vaccine virus for the treatment of cholangiocarcinoma. Hum. Gene Ther..

[118] Zhu Z.B., Chen Y., Makhija S.K., Lu B., Wang M., Rivera A.A., Yamamoto M., Wang S., Siegal G.P., Curiel D.T. (2006). Survivin promoter-based conditionally replicative adenoviruses target cholangiocarcinoma. Int. J. Oncol..

[119] Nelson J.W., Ghafoori A.P., Willett C.G., Tyler D.S., Pappas T.N., Clary B.M., Hurwitz H.I., Bendell J.C., Morse M.A., Clough R.W. (2009). Concurrent chemoradiotherapy in resected extrahepatic cholangiocarcinoma. Int. J. Radiat. Oncol. Biol. Phys..

[120] McMasters K.M., Tuttle T.M., Leach S.D., Rich T., Cleary K.R., Evans D.B., Curley S.A. (1997). Neoadjuvant chemoradiation for extrahepatic cholangiocarcinoma. Am. J. Surg..

[121] Abdelrahim M., Esmail A., He A.R., Khushman M.d., Rayyan Y. (2024). Advances in Immunotherapy for Transplant Oncology. Cancers.

[122] Esmail A., Badheeb M., Alnahar B., Almiqlash B., Sakr Y., Khasawneh B., Al-Najjar E., Al-Rawi H., Abudayyeh A., Rayyan Y. (2024). Cholangiocarcinoma: The Current Status of Surgical Options including Liver Transplantation. Cancers.

[123] Cambridge W.A., Fairfield C., Powell J.J., Harrison E.M., Søreide K., Wigmore S.J., Guest R.V. (2021). Meta-analysis and Meta-regression of Survival After Liver Transplantation for Unresectable Perihilar Cholangiocarcinoma. Ann. Surg..

[124] Yadav S., Xie H., Bin-Riaz I., Sharma P., Durani U., Goyal G., Borah B., Borad M.J., Smoot R.L., Roberts L.R. (2019). Neoadjuvant vs. adjuvant chemotherapy for cholangiocarcinoma: A propensity score matched analysis. Eur. J. Surg. Oncol..

[125] Abdelrahim M., Esmail A., Abudayyeh A., Murakami N., Victor D., Kodali S., Cheah Y.L., Simon C.J., Noureddin M., Connor A. (2023). Transplant Oncology: An Emerging Discipline of Cancer Treatment. Cancers.

[126] Abdelrahim M., Esmail A., Xu J., Umoru G., Al-Rawi H., Saharia A. (2022). P-168 Combination of gemcitabine plus cisplatin compared to non-gemcitabine and cisplatin regimens as neo-adjuvant treatment in liver transplant recipients with cholangiocarcinoma. Ann. Oncol..

[127] Abdelrahim M., Esmail A., Xu J., Umoru G., Al-Rawi H., Saharia A., Abudayyeh A., Victor D., McMillan R., Kodali S. (2022). Gemcitabine plus cisplatin versus non-gemcitabine and cisplatin regimens as neoadjuvant treatment for cholangiocarcinoma patients prior to liver transplantation: An institution experience. Front. Oncol..

[128] Esmail A., Xu J., Umoru G., Al-Rawi H., Saharia A., Abdelrahim M. (2022). P-169 Feasibility of gemcitabine plus cisplatin as neo-adjuvant in cholangiocarcinoma patients prior to liver transplantation. Ann. Oncol..

[129] Abdelrahim M., Al-Rawi H., Esmail A., Xu J., Umoru G., Ibnshamsah F., Abudayyeh A., Victor D., Saharia A., McMillan R. (2022). Gemcitabine and Cisplatin as Neo-Adjuvant for Cholangiocarcinoma Patients Prior to Liver Transplantation: Case-Series. Curr. Oncol..

[130] Maithel S.K., Keilson J.M., Cao H.S.T., Rupji M., Mahipal A., Lin B.S., Javle M.M., Cleary S.P., Akce M., Switchenko J.M. (2023). NEO-GAP: A Single-Arm, Phase II Feasibility Trial of Neoadjuvant Gemcitabine, Cisplatin, and Nab-Paclitaxel for Resectable, High-Risk Intrahepatic Cholangiocarcinoma. Ann. Surg. Oncol..

[131] Hasegawa S., Ikai I., Fujii H., Hatano E., Shimahara Y. (2007). Surgical resection of hilar cholangiocarcinoma: Analysis of survival and postoperative complications. World J. Surg..

[132] Jarnagin W.R., Ruo L., Little S.A., Klimstra D., D’Angelica M., DeMatteo R.P., Wagman R., Blumgart L.H., Fong Y. (2003). Patterns of initial disease recurrence after resection of gallbladder carcinoma and hilar cholangiocarcinoma: Implications for adjuvant therapeutic strategies. Cancer.

[133] Nakachi K., Ikeda M., Konishi M., Mizusawa J., Katayama H., Sano Y., Uesaka K., Yanagimoto H., Morinaga S., Wada H. (2024). Adjuvant S-1 vs. Observation for Resected Biliary Tract Cancer: 5-Year Follow-Up of the JCOG1202/ASCOT.

[134] Nakachi K., Ikeda M., Konishi M., Nomura S., Katayama H., Kataoka T., Todaka A., Yanagimoto H., Morinaga S., Kobayashi S. (2023). Adjuvant S-1 compared with observation in resected biliary tract cancer (JCOG1202, ASCOT): A multicentre, open-label, randomised, controlled, phase 3 trial. Lancet.

[135] Horgan A.M., Amir E., Walter T., Knox J.J. (2012). Adjuvant therapy in the treatment of biliary tract cancer: A systematic review and meta-analysis. J. Clin. Oncol..

[136] Shroff R.T., Kennedy E.B., Bachini M., Bekaii-Saab T., Crane C., Edeline J., El-Khoueiry A., Feng M., Katz M.H.G., Primrose J. (2019). Adjuvant Therapy for Resected Biliary Tract Cancer: ASCO Clinical Practice Guideline. J. Clin. Oncol..

[137] Ben-Josef E., Guthrie K.A., El-Khoueiry A.B., Corless C.L., Zalupski M.M., Lowy A.M., Thomas C.R., Alberts S.R., Dawson L.A., Micetich K.C. (2015). SWOG S0809: A Phase II Intergroup Trial of Adjuvant Capecitabine and Gemcitabine Followed by Radiotherapy and Concurrent Capecitabine in Extrahepatic Cholangiocarcinoma and Gallbladder Carcinoma. J. Clin. Oncol..

[138] Neoptolemos J.P., Moore M.J., Cox T.F., Valle J.W., Palmer D.H., McDonald A.C., Carter R., Tebbutt N.C., Dervenis C., Smith D. (2012). Effect of adjuvant chemotherapy with fluorouracil plus folinic acid or gemcitabine vs observation on survival in patients with resected periampullary adenocarcinoma: The ESPAC-3 periampullary cancer randomized trial. JAMA.

[139] Primrose J.N., Fox R.P., Palmer D.H., Malik H.Z., Prasad R., Mirza D., Anthony A., Corrie P., Falk S., Finch-Jones M. (2019). Capecitabine compared with observation in resected biliary tract cancer (BILCAP): A randomised, controlled, multicentre, phase 3 study. Lancet Oncol..

[140] Park J., Kim M.H., Kim K.P., Park D.H., Moon S.H., Song T.J., Eum J., Lee S.S., Seo D.W., Lee S.K. (2009). Natural History and Prognostic Factors of Advanced Cholangiocarcinoma without Surgery, Chemotherapy, or Radiotherapy: A Large-Scale Observational Study. Gut Liver.

[141] Hare A.E., Makary M.S. (2022). Locoregional Approaches in Cholangiocarcinoma Treatment. Cancers.

[142] Park S.Y., Kim J.H., Yoon H.J., Lee I.S., Yoon H.K., Kim K.P. (2011). Transarterial chemoembolization versus supportive therapy in the palliative treatment of unresectable intrahepatic cholangiocarcinoma. Clin. Radiol..

[143] Gusani N.J., Balaa F.K., Steel J.L., Geller D.A., Marsh J.W., Zajko A.B., Carr B.I., Gamblin T.C. (2008). Treatment of unresectable cholangiocarcinoma with gemcitabine-based transcatheter arterial chemoembolization (TACE): A single-institution experience. J. Gastrointest. Surg..

[144] Kim J.H., Yoon H.-K., Sung K.-B., Ko G.-Y., Gwon D.I., Shin J.H., Song H.-Y. (2008). Transcatheter arterial chemoembolization or chemoinfusion for unresectable intrahepatic cholangiocarcinoma. Cancer.

[145] Boehm L.M., Jayakrishnan T.T., Miura J.T., Zacharias A.J., Johnston F.M., Turaga K.K., Gamblin T.C. (2015). Comparative effectiveness of hepatic artery based therapies for unresectable intrahepatic cholangiocarcinoma. J. Surg. Oncol..

[146] Kemeny N., Daly J., Oderman P., Shike M., Chun H., Petroni G., Geller N. (1984). Hepatic artery pump infusion: Toxicity and results in patients with metastatic colorectal carcinoma. J. Clin. Oncol..

[147] McMillan D.C., McArdle C.S. (2007). Epidemiology of colorectal liver metastases. Surg. Oncol..

[148] Cercek A., Boerner T., Tan B.R., Chou J.F., Gönen M., Boucher T.M., Hauser H.F., Do R.K.G., Lowery M.A., Harding J.J. (2020). Assessment of Hepatic Arterial Infusion of Floxuridine in Combination With Systemic Gemcitabine and Oxaliplatin in Patients With Unresectable Intrahepatic Cholangiocarcinoma: A Phase 2 Clinical Trial. JAMA Oncol..

[149] Cantore M., Mambrini A., Fiorentini G., Rabbi C., Zamagni D., Caudana R., Pennucci C., Sanguinetti F., Lombardi M., Nicoli N. (2005). Phase II study of hepatic intraarterial epirubicin and cisplatin, with systemic 5-fluorouracil in patients with unresectable biliary tract tumors. Cancer.

[150] Ghiringhelli F., Lorgis V., Vincent J., Ladoire S., Guiu B. (2013). Hepatic arterial infusion of gemcitabine plus oxaliplatin as second-line treatment for locally advanced intrahepatic cholangiocarcinoma: Preliminary experience. Chemotherapy.

[151] Ensminger W.D., Gyves J.W. (1983). Clinical pharmacology of hepatic arterial chemotherapy. Semin. Oncol..

[152] Cohen A.D., Kemeny N.E. (2003). An update on hepatic arterial infusion chemotherapy for colorectal cancer. Oncologist.

[153] Higaki T., Aramaki O., Moriguchi M., Nakayama H., Midorikawa Y., Takayama T. (2018). Arterial infusion of cisplatin plus S-1 against unresectable intrahepatic cholangiocarcinoma. Biosci. Trends.

[154] Herber S., Otto G., Schneider J., Manzl N., Kummer I., Kanzler S., Schuchmann A., Thies J., Düber C., Pitton M. (2007). Transarterial chemoembolization (TACE) for inoperable intrahepatic cholangiocarcinoma. Cardiovasc. Interv. Radiol..

[155] Burger I., Hong K., Schulick R., Georgiades C., Thuluvath P., Choti M., Kamel I., Geschwind J.F. (2005). Transcatheter arterial chemoembolization in unresectable cholangiocarcinoma: Initial experience in a single institution. J. Vasc. Interv. Radiol. JVIR.

[156] Liapi E., Geschwind J.F. (2007). Transcatheter and ablative therapeutic approaches for solid malignancies. J. Clin. Oncol..

[157] Stark S., Wang C., Savic L.J., Letzen B., Schobert I., Miszczuk M., Murali N., Oestmann P., Gebauer B., Lin M. (2020). Automated feature quantification of Lipiodol as imaging biomarker to predict therapeutic efficacy of conventional transarterial chemoembolization of liver cancer. Sci. Rep..

[158] Kiefer M.V., Albert M., McNally M., Robertson M., Sun W., Fraker D., Olthoff K., Christians K., Pappas S., Rilling W. (2011). Chemoembolization of intrahepatic cholangiocarcinoma with cisplatinum, doxorubicin, mitomycin C, ethiodol, and polyvinyl alcohol: A 2-center study. Cancer.

[159] Wang L., Lin Z.G., Ke Q., Lou J.Y., Zheng S.G., Bi X.Y., Wang J.M., Guo W., Li F.Y., Wang J. (2020). Adjuvant transarterial chemoembolization following radical resection for intrahepatic cholangiocarcinoma: A multi-center retrospective study. J. Cancer.

[160] Zheng W.H., Yu T., Luo Y.H., Wang Y., Liu Y.F., Hua X.D., Lin J., Ma Z.H., Ai F.L., Wang T.L. (2019). Clinical efficacy of gemcitabine and cisplatin-based transcatheter arterial chemoembolization combined with radiotherapy in hilar cholangiocarcinoma. World J. Gastrointest. Oncol..

[161] Hong K., Khwaja A., Liapi E., Torbenson M.S., Georgiades C.S., Geschwind J.F. (2006). New intra-arterial drug delivery system for the treatment of liver cancer: Preclinical assessment in a rabbit model of liver cancer. Clin. Cancer Res..

[162] Sun J., Zhou G., Zhang Y., Zhou T., Nie C., Zhu T., Chen S., Wang B., Yu Z., Wang H. (2018). Comprehensive analysis of common safety profiles and their predictive factors in 520 records of liver cancer patients treated by drug-eluting beads transarterial chemoembolization. Medicine.

[163] Poggi G., Amatu A., Montagna B., Quaretti P., Minoia C., Sottani C., Villani L., Tagliaferri B., Sottotetti F., Rossi O. (2009). OEM-TACE: A new therapeutic approach in unresectable intrahepatic cholangiocarcinoma. Cardiovasc. Interv. Radiol..

[164] Luo J., Zheng J., Shi C., Fang J., Peng Z., Huang J., Sun J., Zhou G., Li T., Zhu D. (2020). Drug-eluting beads transarterial chemoembolization by CalliSpheres is effective and well tolerated in treating intrahepatic cholangiocarcinoma patients: A preliminary result from CTILC study. Medicine.

[165] Hori A., Toei H., Nakamura T., Makitani K., Hori S. (2022). Successful control of a large intrahepatic cholangiocarcinoma treated by transarterial chemo-embolization; a single case report. BJR Case Rep..

[166] Gates V.L., Atassi B., Lewandowski R.J., Ryu R.K., Sato K.T., Nemcek A.A., Omary R., Salem R. (2007). Radioembolization with Yttrium-90 microspheres: Review of an emerging treatment for liver tumors. Future Oncol..

[167] Ettorre G.M., Levi Sandri G.B., Laurenzi A., Colasanti M., Meniconi R.L., Lionetti R., Santoro R., Lepiane P., Sciuto R., Pizzi G. (2017). Yttrium-90 Radioembolization for Hepatocellular Carcinoma Prior to Liver Transplantation. World J. Surg..

[168] Mahnken A.H. (2016). Current status of transarterial radioembolization. World J. Radiol..

[169] Houle S., Yip T.K., Shepherd F.A., Rotstein L.E., Sniderman K.W., Theis E., Cawthorn R.H., Richmond-Cox K. (1989). Hepatocellular carcinoma: Pilot trial of treatment with Y-90 microspheres. Radiology.

[170] Ibrahim S.M., Mulcahy M.F., Lewandowski R.J., Sato K.T., Ryu R.K., Masterson E.J., Newman S.B., Benson A., Omary R.A., Salem R. (2008). Treatment of unresectable cholangiocarcinoma using yttrium-90 microspheres: Results from a pilot study. Cancer.

[171] Sarwar A., Ali A., Ljuboja D., Weinstein J.L., Shenoy-Bhangle A.S., Nasser I.A., Morrow M.K., Faintuch S., Curry M.P., Bullock A.J. (2021). Neoadjuvant Yttrium-90 Transarterial Radioembolization with Resin Microspheres Prescribed Using the Medical Internal Radiation Dose Model for Intrahepatic Cholangiocarcinoma. J. Vasc. Interv. Radiol..

[172] Chan S.L., Chotipanich C., Choo S.P., Kwang S.W., Mo F., Worakitsitisatorn A., Tai D., Sundar R., Ng D.C.E., Loke K.S.H. (2022). Selective Internal Radiation Therapy with Yttrium-90 Resin Microspheres Followed by Gemcitabine plus Cisplatin for Unresectable Intrahepatic Cholangiocarcinoma: A Phase 2 Single-Arm Multicenter Clinical Trial. Liver Cancer.

[173] Zhou T.Y., Zhou G.H., Zhang Y.L., Nie C.H., Zhu T.Y., Wang H.L., Chen S.Q., Wang B.Q., Yu Z.N., Wu L.M. (2020). Drug-eluting beads transarterial chemoembolization with CalliSpheres microspheres for treatment of unresectable intrahepatic cholangiocarcinoma. J. Cancer.

[174] Aliberti C., Carandina R., Sarti D., Pizzirani E., Ramondo G., Mulazzani L., Mattioli G.M., Fiorentini G. (2017). Chemoembolization with Drug-eluting Microspheres Loaded with Doxorubicin for the Treatment of Cholangiocarcinoma. Anticancer. Res..

[175] Hong K., Georgiades C. (2010). Radiofrequency ablation: Mechanism of action and devices. J. Vasc. Interv. Radiol. JVIR.

[176] Tuttle R., Nurkin S.J., Hochwald S.N. (2014). Ablative therapy for esophageal dysplasia and early malignancy: Focus on RFA. BioMed Res. Int..

[177] Tanabe K.K., Kulu Y. (2007). Radiofrequency Ablation for Colon and Rectal Carcinoma Liver Metastases: What’s Missing?. Gastrointest. Cancer Res. GCR.

[178] Han K., Ko H.K., Kim K.W., Won H.J., Shin Y.M., Kim P.N. (2015). Radiofrequency ablation in the treatment of unresectable intrahepatic cholangiocarcinoma: Systematic review and meta-analysis. J. Vasc. Interv. Radiol. JVIR.

[179] Kim J.H., Won H.J., Shin Y.M., Kim K.A., Kim P.N. (2011). Radiofrequency ablation for the treatment of primary intrahepatic cholangiocarcinoma. AJR Am. J. Roentgenol..

[180] Giorgio A., Calisti G., De Stefano G., Farella N., Di Sarno A., Amendola F., Scognamiglio U., Giorgio V. (2011). Radiofrequency ablation for intrahepatic cholangiocarcinoma: Retrospective analysis of a single centre experience. Anticancer. Res..

[181] Brandi G., Rizzo A., Dall’Olio F.G., Felicani C., Ercolani G., Cescon M., Frega G., Tavolari S., Palloni A., De Lorenzo S. (2020). Percutaneous radiofrequency ablation in intrahepatic cholangiocarcinoma: A retrospective single-center experience. Int. J. Hyperth..

[182] Wu T.T., Li W.M., Li H.C., Ao G.K., Zheng F., Lin H. (2017). Percutaneous Intraductal Radiofrequency Ablation for Extrahepatic Distal Cholangiocarcinoma: A Method for Prolonging Stent Patency and Achieving Better Functional Status and Quality of Life. Cardiovasc. Interv. Radiol..

[183] Davalos R.V., Mir I.L., Rubinsky B. (2005). Tissue ablation with irreversible electroporation. Ann. Biomed. Eng..

[184] Tarek M. (2005). Membrane electroporation: A molecular dynamics simulation. Biophys. J..

[185] Delemotte L., Tarek M. (2012). Molecular dynamics simulations of lipid membrane electroporation. J. Membr. Biol..

[186] Belfiore M.P., Reginelli A., Maggialetti N., Carbone M., Giovine S., Laporta A., Urraro F., Nardone V., Grassi R., Cappabianca S. (2020). Preliminary results in unresectable cholangiocarcinoma treated by CT percutaneous irreversible electroporation: Feasibility, safety and efficacy. Med. Oncol..

[187] Martin E.K., Bhutiani N., Egger M.E., Philips P., Scoggins C.R., McMasters K.M., Kelly L.R., Vitale G.C., Martin R.C.G. (2018). Safety and efficacy of irreversible electroporation in the treatment of obstructive jaundice in advanced hilar cholangiocarcinoma. HPB.

[188] Franken L.C., van Veldhuisen E., Ruarus A.H., Coelen R.J.S., Roos E., van Delden O.M., Besselink M.G., Klümpen H.J., van Lienden K.P., van Gulik T.M. (2022). Outcomes of Irreversible Electroporation for Perihilar Cholangiocarcinoma: A Prospective Pilot Study. J. Vasc. Interv. Radiol. JVIR.

[189] Owen M., Makary M.S., Beal E.W. (2023). Locoregional Therapy for Intrahepatic Cholangiocarcinoma. Cancers.

[190] Rubinsky B., Lee C.Y., Bastacky J., Onik G. (1990). The process of freezing and the mechanism of damage during hepatic cryosurgery. Cryobiology.

[191] Glazer D.I., Tatli S., Shyn P.B., Vangel M.G., Tuncali K., Silverman S.G. (2017). Percutaneous Image-Guided Cryoablation of Hepatic Tumors: Single-Center Experience With Intermediate to Long-Term Outcomes. AJR Am. J. Roentgenol..

[192] Valle J.W., Borbath I., Khan S.A., Huguet F., Gruenberger T., Arnold D. (2016). Biliary cancer: ESMO Clinical Practice Guidelines for diagnosis, treatment and follow-up. Ann. Oncol..

[193] Jethwa K.R., Sannapaneni S., Mullikin T.C., Harmsen W.S., Petersen M.M., Antharam P., Laughlin B., Mahipal A., Halfdanarson T.R., Merrell K.W. (2020). Chemoradiotherapy for patients with locally advanced or unresectable extra-hepatic biliary cancer. J. Gastrointest. Oncol..

[194] Nag S., DeHaan M., Scruggs G., Mayr N., Martin E.W. (2006). Long-term follow-up of patients of intrahepatic malignancies treated with iodine-125 brachytherapy. Int. J. Radiat. Oncol. Biol. Phys..

[195] Zhu H.D., Guo J.H., Huang M., Ji J.S., Xu H., Lu J., Li H.L., Wang W.H., Li Y.L., Ni C.F. (2018). Irradiation stents vs. conventional metal stents for unresectable malignant biliary obstruction: A multicenter trial. J. Hepatol..

[196] Wang T., Liu S., Zheng Y.B., Song X.P., Sun B.L., Jiang W.J., Wang L.G. (2017). Clinical Study on Using (125)I Seeds Articles Combined with Biliary Stent Implantation in the Treatment of Malignant Obstructive Jaundice. Anticancer. Res..

[197] Shinohara E.T., Guo M., Mitra N., Metz J.M. (2010). Brachytherapy in the treatment of cholangiocarcinoma. Int. J. Radiat. Oncol. Biol. Phys..

[198] Zhu H.D., Guo J.H., Zhu G.Y., He S.C., Fang W., Deng G., Qin Y.L., Li G.Z., Coldwell D.M., Teng G.J. (2012). A novel biliary stent loaded with (125)I seeds in patients with malignant biliary obstruction: Preliminary results versus a conventional biliary stent. J. Hepatol..

[199] Khosla D., Zaheer S., Gupta R., Madan R., Goyal S., Kumar N., Kapoor R. (2022). Role of intraluminal brachytherapy in palliation of biliary obstruction in cholangiocarcinoma: A brief review. World J. Gastrointest. Endosc..

[200] Baak R., Willemssen F., van Norden Y., Eskens F., Milder M.T.W., Heijmen B.J.M., Koerkamp B.G., Sprengers D., van Driel L., Klümpen H.J. (2021). Stereotactic Body Radiation Therapy after Chemotherapy for Unresectable Perihilar Cholangiocarcinoma: The STRONG Trial, a Phase I Safety and Feasibility Study. Cancers.

[201] Hong T.S., Wo J.Y., Yeap B.Y., Ben-Josef E., McDonnell E.I., Blaszkowsky L.S., Kwak E.L., Allen J.N., Clark J.W., Goyal L. (2016). Multi-Institutional Phase II Study of High-Dose Hypofractionated Proton Beam Therapy in Patients With Localized, Unresectable Hepatocellular Carcinoma and Intrahepatic Cholangiocarcinoma. J. Clin. Oncol..

[202] Smart A.C., Goyal L., Horick N., Petkovska N., Zhu A.X., Ferrone C.R., Tanabe K.K., Allen J.N., Drapek L.C., Qadan M. (2020). Hypofractionated Radiation Therapy for Unresectable/Locally Recurrent Intrahepatic Cholangiocarcinoma. Ann. Surg. Oncol..

[203] De B., Upadhyay R., Liao K., Kumala T., Shi C., Dodoo G., Abi Jaoude J., Corrigan K.L., Manzar G.S., Marqueen K.E. (2023). Definitive Liver Radiotherapy for Intrahepatic Cholangiocarcinoma with Extrahepatic Metastases. Liver Cancer.

